# Plasma‐Assisted Reforming of Methane

**DOI:** 10.1002/advs.202203221

**Published:** 2022-10-17

**Authors:** Jiayu Feng, Xin Sun, Zhao Li, Xingguang Hao, Maohong Fan, Ping Ning, Kai Li

**Affiliations:** ^1^ Faculty of Environmental Science and Engineering Kunming University of Science and Technology Kunming 650500 P. R. China; ^2^ Departments of Chemical and Petroleum Engineering University of Wyoming Laramie WY 82071 USA; ^3^ School of Energy Resources University of Wyoming Laramie WY 82071 USA; ^4^ School of Civil & Environmental Engineering Georgia Institute of Technology Atlanta GA 30332 USA

**Keywords:** catalyst, greenhouse gas, methane, plasma, reforming

## Abstract

Methane (CH_4_) is inexpensive, high in heating value, relatively low in carbon footprint compared to coal, and thus a promising energy resource. However, the locations of natural gas production sites are typically far from industrial areas. Therefore, transportation is needed, which could considerably increase the sale price of natural gas. Thus, the development of distributed, clean, affordable processes for the efficient conversion of CH_4_ has increasingly attracted people's attention. Among them are plasma technology with the advantages of mild operating conditions, low space need, and quick generation of energetic and chemically active species, which allows the reaction to occur far from the thermodynamic equilibrium and at a reasonable cost. Significant progress in plasma‐assisted reforming of methane (PARM) is achieved and reviewed in this paper from the perspectives of reactor development, thermal and nonthermal PARM routes, and catalysis. The factors affecting the conversion of reactants and the selectivity of products are studied. The findings from the past works and the insight into the existing challenges in this work should benefit the further development of reactors, high‐performance catalysts, and PARM routes.

## Graphical abstract

Plasma‐assisted reforming of methane (PARM) is a potential methane conversion strategy, which can overcome the thermodynamic limitations and make the methane reforming process proceed under relatively mild conditions.

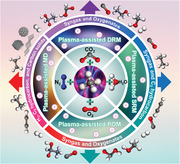

## 1 Introduction

CH_4_ (primarily from natural gas, CH_4_ hydrate, and shale gas) is a widely available and underutilized feedstock with increasing proven reserves.^[^ ^1^ ^]^ It could satisfy the global energy requirements for heating, transportation, and power generation.^[^ ^2^ ^]^ CH_4_ is the simplest organic substance and the hydrocarbon with the least carbon content (most hydrogen content). Its low‐cost transportation is a challenge due to its low boiling point (161.5 °C) and high flammability. In general, CH_4_ is commonly burned as flare gas; as a result, approximately 140 billion m^3^ of CH_4_ (≈$20 billion) is wasted every year.^[^ ^3^ ^]^ Furthermore, approximately 8% of CH_4_ diffuses into the atmosphere, contributing to global warming (25 times more potent than CO_2_).^[^ ^4^, ^5^ ^]^ Many researchers have focused on transforming CH_4_ into more energy‐dense fuels or higher value‐added chemicals to solve this problem.

However, the CH_4_ transformation process is limited owing to the strong C—H bond (434 kJ mol^−1^),^[^ ^6^ ^]^ which implies that dehydrogenation (H‐extraction) requires high energy input. Additionally, the CH_4_ molecule has a symmetrical tetrahedral geometry and weakly polarized C—H bonds, which lead to its weak acidity (p*K*a = 40), making CH_4_ more resistant to deprotonation reactions.^[^ ^7^ ^]^ The high stability of CH_4_ can also be further demonstrated by the electron affinity (activation by nucleophiles) of 145 kJ mol^−1^, proton affinity (activation by protons) of 552 kJ mol^−1^, and ionization potential (activation by removal of electrons) of 1215 kJ mol^−1^. There are four types of routes for CH_4_ transformation: partial oxidation of methane (POM), catalytic decomposition of methane (CDM), steam methane reforming (SMR), and dry reforming of methane (DRM).^[^ ^8^ ^]^ At present, CH_4_ can be converted to more energy‐dense fuels or value‐added chemicals by various techniques such as conventional catalytic technology, plasma technology, thermal pyrolysis, and biotechnology. Conventional catalytic technologies have been extensively studied owing to their high selectivity and yield; however, numerous problems (catalyst poisoning, carbon deposition, coking, and sintering caused by high reaction temperatures) limit their application. Simultaneously, thermal pyrolysis is an energy‐consuming process that produces fewer products than plasma.^[^ ^9^, ^10^, ^11^, ^12^ ^]^ Biotechnology is also a promising method. However, the preparation of screening the bacterial culture and moderate temperatures limits the application of this technology in the industry. In recent years, plasma‐assisted methane reforming (PARM) technology has received extensive attention due to its potential advantages of mild reaction conditions, avoidance of catalyst coking, compact reactor size, and fast response time.^[^ ^13^ ^]^

**Figure 1:**
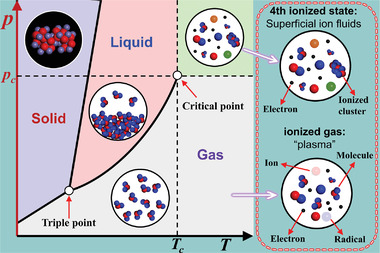
Schematic diagram of corresponding ionized states of matter in different phases. Reproduced under the terms of a Creative Commons Attribution 3.0 International License.^[^ ^17^ ^]^ Copyright 2017, IOP Publishing Ltd.

The concept of “plasma” was first proposed by Irving Langmuir in 1928.^[^ ^14^ ^]^ Plasma represents a special conductive fluid (ionized gas or sometimes liquid) composed of ions and positive/negative electrons, neutral/excited/ground‐state atoms/molecules, free radicals, and photons.^[^ ^15^, ^16^ ^]^ A typical feature of plasma is that the entire system is electrically neutral whether it is partially or completely ionized. Plasma is different from the other three basic states of matter, namely solid, liquid and gas, and it is the fourth state of a substance (**Figure** **1**).^[^ ^17^ ^]^ Broadly, plasma generally refers to fully or partially ionized gas, which can be divided into two types according to the temperature: high‐temperature plasma (such as corona/stellar plasma) and low‐temperature plasma.^[^ ^18^, ^19^ ^]^ Low‐temperature plasma is traditionally divided into thermal plasma and non‐thermal plasma.^[^ ^20^ ^]^ Thermal plasmas include arcs, plasma jets, and plasma torches, in which various particles are in thermal equilibrium with temperatures as high as tens of eV (1 eV = 11 605 K/11 331.85 °C).^[^ ^21^, ^22^ ^]^ Conversely, nonthermal plasmas (cold plasmas) consist of nonequilibrium ionized gases with temperatures at room temperature or even lower and electron temperatures as low as −168 °C.^[^ ^23^, ^24^ ^]^ A typical nonthermal plasma consists of mercury vapor gas inside a fluorescent lamp, which can be touched while operating due to its lower temperature. Based on the type of reactor, power supply, electrode configuration, and discharge parameter configuration,^[^ ^25^ ^]^ different plasmas can be obtained, such as corona discharges,^[^ ^26^ ^]^ spark discharges,^[^ ^27^ ^]^ radio frequency (RF) discharges, gliding arc discharges (GAD),^[^ ^28^ ^]^ dielectric barrier discharges (DBDs),^[^ ^29^ ^]^ microwaves (MW) discharge,^[^ ^30^ ^]^ and alternative current (AC) glow discharges.^[^ ^31^ ^]^ Three key features of plasma technology are attractive for methane reforming: I) the temperature and energy densities of the components in some plasma phases can greatly exceed those of conventional catalytic processes; II) the plasma can provide high concentrations of energetic chemically active particles to facilitate the reaction; and III) plasma systems can essentially be far from thermodynamic equilibrium, allowing the chemical reaction to proceed under relatively mild conditions.^[^ ^32^ ^]^ These features allow PARM technology to overcome some of the shortcomings of conventional methane reforming techniques. PARM is generally considered a potential route to convert CH_4_ into chemical products and fuels at near‐ambient temperatures and pressures, especially for distributed processes based on renewable energy sources.^[^ ^33^ ^]^ Additionally, the synergistic effect of plasma and a catalyst can further improve the efficiency of CH_4_ reforming.^[^ ^34^ ^]^ However, in the PARM process, it is not only necessary to construct suitable catalysts but also to find appropriate states and optimal plasma discharge parameters among the numerous possibilities inherent in systems far from thermodynamic equilibrium.^[^ ^32^ ^]^ Among these parameters, the reactor type (discharge type) has a significant effect on the progress of PARM.

Current developments in plasma‐assisted methane reforming technology must be rationally understood. Therefore, this review emphasizes introducing the application of plasma technology to different CH_4_ reforming routes and the application of catalysts in PARM systems. After an in‐depth discussion of the basic concepts and general reaction mechanisms of PARM, a critical discussion on the recent progress in reactor design and catalyst construction for the PARM system is provided with a clear classification of the PARM routes. In addition, PARM technology's challenges and our outlook for future research directions are presented to guide scientific research and industrial applications. We look forward to analyzing and summarizing the literature selected in this review, providing recommendations for the research and development of PARM while promoting the application of plasma technology in the reforming of CH_4_.

## 2 Fundamentals of Plasma‐Assisted Reforming of Methane

### 2.1 Conventional Thermocatalytic Reforming of CH_4_

**Figure 2:**
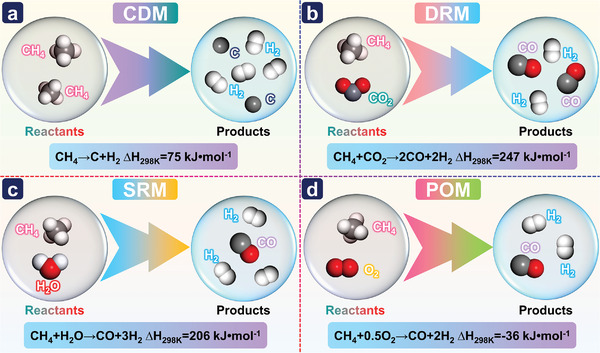
Four common CH_4_ reforming reactions: a) CDM, b) DRM, c) SMR, and d) POM.^[^ ^36^ ^]^

The conversion of CH_4_ to more valuable chemicals is one of the most intensively studied topics in the field of catalysis. Currently, in industry, CH_4_ is converted to syngas (CO+H_2_) at high temperatures through an indirect route, which is then used to produce valuable hydrocarbons or alcohols. This is an energy‐intensive and costly process, so lowering the reaction temperature for CH_4_ conversion is beneficial. However, the direct conversion of CH_4_ to derivatives, which is thermodynamically feasible but kinetically difficult, creates more challenges for CH_4_ conversion.^[^ ^35^ ^]^ Nonetheless, considerable effort has been devoted to CH_4_ conversion to produce the desired chemicals in high yields. CH_4_ reforming technology has undergone nearly a century of development. Commonly used conventional CH_4_ reforming processes include CDM, DRM, SMR, and POM, as shown in **Figure** **2**.

#### 2.1.1 CDM

CDM is a CO*_x_*‐free one‐step H_2_ production method,^[^ ^37^ ^]^ and its reaction mechanism involves the production of pure H_2_ and carbon solids by the catalytic decomposition of CH_4_. Consequently, greenhouse gas emissions (CO or CO_2_) are virtually eliminated. The reaction is represented by R2.1,^[^ ^38^, ^39^ ^]^ and its total energy and heat requirements are much lower than those of the SMR and DRM processes,^[^ ^8^ ^]^ which has valuable implications for reducing the operating temperature and equipment operating costs. In addition, the CDM process can directly obtain pure H_2_ without considering by‐product separation and CO_2_ emissions.^[^ ^39^, ^40^ ^]^ Some studies have demonstrated that the carbon solid produced in the CDM process can also serve as a valuable by‐product to improve the economics of the system, for example, in the form of carbon nanotubes.^[^ ^41^, ^42^ ^]^ The catalyst can effectively tune the CDM process's H_2_ yield and carbon structure.^[^ ^37^ ^]^ Abbas and Daud^[^ ^43^ ^]^ reviewed the development of metal and carbonaceous CDM catalysts. According to them, by far the most studied carbon‐containing catalysts are activated carbon and carbon black, while the most common metal catalysts are nickel (Ni), iron (Fe), and copper (Cu). We also noticed a series of reports on the use of molten metals/salts as CDM catalysts.^[^ ^44^, ^45^, ^46^, ^47^ ^]^ Upham et al.^[^ ^48^ ^]^ pointed out that active metals (Ni, Pt, Pd) dissolved in inactive low melting point metals (in, Ga, Sn, Pb) produce stable molten metal alloy catalysts that can be used for the pyrolysis of CH_4_ to H_2_ and carbon. The carbon produced by the decomposition of CH_4_ floats to the surface of the molten metals/salts catalyst and can be easily removed, thus effectively avoiding catalyst deactivation due to coking.^[^ ^48^, ^49^ ^]^ Furthermore, this molten metals/salts catalytic pyrolysis CH_4_ system has significant advantages for CO_2_‐free H_2_ production and may even represent the most cost‐effective method for CO_2_‐free H_2_ and competes favorably to SMR with carbon capture and sequestration.^[^ ^50^, ^51^ ^]^ Mondal and Chandran^[^ ^52^ ^]^ conducted an economic and environmental analysis of the CDM process and concluded that the operating temperature of the CDM process could be lower than that of the SMR process, which would result in significant energy savings. Weger et al.^[^ ^53^ ^]^ evaluated the hydrogen economy in the practical implementation of the CDM process and pointed out that the CDM process could be a bridge to a sustainable hydrogen economy. Although CDM has the above advantages, it also suffers from a fatal problem: carbon deposition leads to catalyst deactivation.^[^ ^8^, ^54^ ^]^ Recent research has focused on the development of a low‐cost, high‐activity, and long‐life catalyst for CDM processes that effectively overcomes the adverse effects of carbon deposition. CDM may be an advantageous technology suitable for small to medium‐scale industrial H_2_ production, and alloy catalysts are considered a promising route for CDM commercialization.^[^ ^38^ ^]^

(R2.1) $$\mathrm{CDM}:\;{\mathrm{CH}}_{4}\to C+2H_{2}\;\Delta H_{298K}=75\;\mathrm{kJ}\;{\mathrm{mol}}^{-1}$$

#### 2.1.2 DRM

The reforming of CH_4_ using CO_2_ is called DRM. The DRM process can convert CH_4_ and CO_2_ (the main components of greenhouse gases) into syngas, which is suitable for Fischer‐Tropsch synthesis (FTS) and is expressed as the reaction R2.2.^[^ ^55^, ^56^ ^]^

(R2.2) $$\begin{array}{c} \mathrm{DRM}:\;{\mathrm{CH}}_{4}+{\mathrm{CO}}_{2}\to 2\mathrm{CO}+2H_{2}\;\Delta H_{298K}=247\;\mathrm{kJ}\;{\mathrm{mol}}^{-1} \end{array}$$

The reaction proceeds at high temperatures and is 20% more endothermic than SMR.^[^ ^57^, ^58^ ^]^ The DRM process is an attractive strategy for CH_4_ reforming. It can alleviate environmental problems such as greenhouse gas emissions and produce value‐added syngas as a chemical feedstock, which is vital in reducing carbon emissions. However, since both CH_4_ and CO_2_ contain carbon, carbon deposition is inevitable in the PADRM process via the following thermodynamic reactions:^[^ ^31^, ^59^ ^]^

(R2.3) $${\mathrm{CH}}_{4}\to C+2H_{2}\Delta H_{298K}=75\mathrm{kJ}{\mathrm{mol}}^{-1}$$

(R2.4) $${\mathrm{CO}}_{2}+2H_{2}\to C+2H_{2}O\;\Delta H_{298K}=-90\;\mathrm{kJ}\;{\mathrm{mol}}^{-1}$$

(R2.5) $$2\mathrm{CO}\to C+{\mathrm{CO}}_{2}\;\Delta H_{298K}=-172.4\;\mathrm{kJ}\;{\mathrm{mol}}^{-1}$$

(R2.6) $$\mathrm{CO}+H_{2}\to C+H_{2}O\;\Delta H_{298K}=-131\;\mathrm{kJ}\;{\mathrm{mol}}^{-1}$$

Reaction R2.3 is CH_4_ cracking or pyrolysis; Reaction R2.4 involves H_2_O formation via CO_2_ reforming; Reaction R2.5 is the Boudouard reaction,^[^ ^60^ ^]^ whereas Reaction R2.6 is the “Reverse Water Gas Shift (RWGS).”^[^ ^61^, ^62^ ^]^ For the enthalpy change of each reaction, Reaction R2.3 is endothermic, and the other reactions are exothermic. In other words, high temperature is conducive to the formation of carbon deposition. Carbon deposition can lead to catalyst deactivation. Temperatures above 900 °C are generally required to completely prevent the formation of carbon deposits. Compared with noble metals (Rh, Ru, and Pt), bimetallic (Ni–Pt, Ni–Rh, Ni–Ce, Ni–Mo, Ni–Co) and monometallic (Ni) catalysts are preferred for DRM because of their low cost,^[^ ^63^, ^64^, ^65^ ^]^ and the addition of potassium to the catalyst can suppress coke at relatively low temperatures. In future DRM catalyst research, Wang et al.^[^ ^66^ ^]^ suggest that low‐temperature DRM catalysts may be made possible by investigating the interaction of supports, promoters with nickel, as well as their structural adjustment. Moreover, the direct conversion of CO_2_ and CH_4_ to liquid oxygenates (e.g., acetic acid) is another promising route for CO_2_ resource and CH_4_ activation.^[^ ^67^, ^68^ ^]^ Since the atom economy is 100% reaction (R2.7), this is an attractive route. Recently, Zhao et al.^[^ ^69^ ^]^ investigated the direct reaction of CO_2_ and CH_4_ to generate CH_3_COOH over zinc‐doped ceria catalyst using theoretical calculation modeling (DFT), zinc‐doped ceria catalyst was found to catalyze the co‐conversion of CH_4_ and CO_2_, while CH_3_COOH was generated through C—C coupling on the catalyst surface. However, the reaction R2.7 is thermodynamically unfavorable under practical conditions. It is almost impossible to convert CO_2_ and CH_4_ into liquid oxygenates in a one‐step catalytic process bypassing syngas production (R2.2). It has been reported that catalysts such as Cu/Co‐based catalysts, Pd/C, Pt/Al_2_O_3_, Pd/SiO_2_, and Rh/SiO_2_ can convert CO_2_ and CH_4_ to CH_3_COOH by step‐wise heterogeneous catalysis.^[^ ^67^, ^70^, ^71^, ^72^ ^]^ These catalysts first contact CH_4_ and form CH*_x_* species on the surface. Subsequently, the feed gas changes from CH_4_ to CO_2,_ and CO_2_ reacts with CH*_x_* on the catalysts to produce CH_3_COOH. Although this is a complex process, it offers more possibilities for DRM systems. DRM has considerable environmental potential, but it is an industrially immature process. In addition, high capital investment and limited commercial experience limit further development.

(R2.7) $$\begin{array}{c} \begin{array}{c} \mathrm{DRM}\;\left(\mathrm{gas}\;\mathrm{to}\;\mathrm{liquid}\right):\;{\mathrm{CH}}_{4}+{\mathrm{CO}}_{2}\to {\mathrm{CH}}_{3}\mathrm{COOH}\;\Delta H_{298K}=71.17\;\mathrm{kJ}\;{\mathrm{mol}}^{-1} \end{array} \end{array}$$

#### 2.1.3 SMR

The SMR reaction produces syngas via the reaction between CH_4_ and water vapor. This endothermic reaction is expressed as reaction R2.8.^[^ ^73^, ^74^ ^]^ In this reaction, CH_4_ and H_2_O are used as hydrogen sources to produce H_2_. Today, over 90% of the world's total H_2_ production is derived from CH_4_,^[^ ^75^ ^]^ and approximately 50% of the global demand for H_2_ is provided by the SMR process,^[^ ^76^ ^]^ since 3 moles or more of H_2_ are produced per mole of CH_4_.^[^ ^52^ ^]^ The SMR process is often used in the fertilizer (H_2_ is used for NH_3_ production) and natural gas industries.^[^ ^77^ ^]^ Ni‐based catalysts are widely used in the SMR process. As early as 1998, Besenbacher et al.^[^ ^78^ ^]^ pointed out that high specific surface area nickel alloy catalysts have great prospects in the SMR process. Sehested et al.^[^ ^79^ ^]^ pointed out that there are four challenges in the future development of Ni‐based SMR catalysts: activity, sulfur poisoning, carbon formation, and sintering. To improve the activity and coking resistance of SMR catalysts, tremendous efforts (such as surface modification, the addition of promoters, catalyst structure optimization, development of high‐performance supports, etc.) have been made in the past decade.^[^ ^80^ ^]^ In addition, noble metal catalysts (Ru, Rh, and Pt) exhibit high activity in the SMR process,^[^ ^81^, ^82^, ^83^, ^84^, ^85^ ^]^ but the high cost limits their further development. Other non‐noble transition metal catalysts, such as Co/Al_2_O_3_,^[^ ^86^ ^]^ Cu/Co_6_A_l2_,^[^ ^87^ ^]^ and Mo_2_C/Al_2_O_3_,^[^ ^87^ ^]^ have shown great potential, but complex synthetic processes may also be an obstacle to its commercialization.

Although SMR is the most widely accepted H_2_ production process, the reaction needs to be carried out at high temperatures due to its kinetic and thermodynamic limitations.^[^ ^88^ ^]^ The SMR process requires a high energy and heat supply, and the reaction system is usually energy intensive.^[^ ^57^, ^89^ ^]^ Therefore, the production of the energy required for the SMR process generates substantial greenhouse gases. Mondal and Chandran^[^ ^52^ ^]^ conducted economic and environmental analysis on two H_2_ production processes, SMR and CDM, and concluded that the SMR process has a higher carbon footprint. Although SMR is a mature process that has been commercialized, or perhaps because of this, its entire industrial process is difficult to meet the recently emerging carbon emission reduction needs, mainly manifested in poor energy integration efficiency.^[^ ^90^, ^91^ ^]^ Therefore, it is very important to make technical improvements to SMR. The SMR process is accompanied by a water‐gas shift (WGS), which is an exothermic reaction expressed as reaction R2.9.^[^ ^74^, ^90^ ^]^ SMR is highly endothermic, while WGS is moderately exothermic. Both reactions are equilibrium restricted. However, if the CO_2_ can be removed from the gas phase, it is possible to make the reactants close to complete conversion. Based on the above theory, to change the normal equilibrium limit of the SMR process, improve the purity of H_2_ (even eliminate the subsequent purification steps of H_2_), and reduce CO_2_ emissions, sorption‐enhanced steam methane reforming (SESMR) process is designed.^[^ ^90^, ^91^, ^92^, ^93^ ^]^ This process removes CO_2_ from the SMR system via an adsorbent, and various CO_2_ absorbers have been reported, including calcium‐based oxides, potassium‐promoted hydrotalcite, and mixed metal oxides of lithium and sodium.^[^ ^91^ ^]^ Since the sorbent is consumed during H_2_ production, the process is inherently unstable. Compared to the SMR process, the SESMR process has many potential advantages, including improved energy efficiency and lower investment costs through process simplification.^[^ ^93^ ^]^ However, the choice of adsorbent should be cautious and objective, and the preparation cost of the adsorbent itself and the regeneration cost of the deactivated adsorbent needs to be considered. In conclusion, to make H_2_ production engineering available and affordable, conventional SMR processes cannot be abandoned yet.^[^ ^74^, ^92^ ^]^

(R2.8) $$\begin{array}{c} \mathrm{SMR}:\;{\mathrm{CH}}_{4}+H_{2}O\to \mathrm{CO}+3H_{2}\;\Delta H_{298K}=206\;\mathrm{kJ}\;{\mathrm{mol}}^{-1} \end{array}$$

(R2.9) $$\begin{array}{c} \mathrm{WGS}:\;\mathrm{CO}+H_{2}O\to {\mathrm{CO}}_{2}+H_{2}\;\Delta H_{298K}=-41.2\;\mathrm{kJ}\;{\mathrm{mol}}^{-1} \end{array}$$

#### 2.1.4 POM

The POM reaction is an exothermic process expressed as reaction R2.10.^[^ ^94^, ^95^, ^96^ ^]^ In the POM reaction, the molar ratio of H_2_ and CO in the product (syngas) is 2, which is different from that of the SMR reaction (H_2_/CO = 3) and the DRM reaction (H_2_/CO = 1). The POM process's coupled exothermic oxidation reaction, and endothermic reforming reactions can be carried out simultaneously in the catalyst bed. Producing syngas through simultaneous exothermic and endothermic reactions is attractive because it significantly reduces the energy requirements.^[^ ^96^ ^]^ Noncatalytic POM occurs only at extremely high temperatures.^[^ ^97^ ^]^ Many studies have focused on the POM reaction over different catalysts.^[^ ^98^, ^99^, ^100^, ^101^, ^102^ ^]^ Although several catalysts have been developed showing activity for the POM reaction, there is still a problem that cannot be ignored: the rapid deactivation of the catalyst due to carbon deposition.^[^ ^103^, ^104^, ^105^, ^106^ ^]^ Increasing the O_2_/CH_4_ ratio and reaction temperature can avoid undesired carbon deposition on the catalyst surface, but it also increases the potential explosion hazard. In addition, direct partial oxidation of CH_4_ to methanol (CH_3_OH) has been attracting significant attention since it was found possible in 1902 because of its great industrial potential for the utilization of natural gas.^[^ ^107^ ^]^ The reactions of CH_4_ to CH_3_OH by direct routes are as R2.11. Conventionally, the liquefaction of natural gas is an indirect process. CH_4_ is first converted to syngas by an SMR process and then catalytically converted to methanol. This is an energy‐intensive process. To reduce the cost, research on the partial oxidation of CH_4_ directly to methanol has been paid attention by researchers.^[^ ^108^, ^109^, ^110^ ^]^ The development of high‐performance and high‐selectivity catalysts is the focus of research in the partial oxidation of CH_4_ to methanol. Although a series of catalysts have been developed to convert CH_4_ to methanol in the laboratory,^[^ ^101^, ^109^, ^110^ ^]^ achieving industrial scale is a challenge.

(R2.10) $$\begin{array}{c} \begin{array}{c} \mathrm{POM}\;\left(\mathrm{syngas}\right):\;{\mathrm{CH}}_{4}+\frac{1}{2}O_{2}\to \mathrm{CO}+2H_{2}↑\;\Delta H_{298K}=-36\;\mathrm{kJ}\;{\mathrm{mol}}^{-1} \end{array} \end{array}$$

(R2.11) $$\begin{array}{c} \begin{array}{c} \mathrm{POM}\;\left(\mathrm{methanol}\right):\;{\mathrm{CH}}_{4}+\frac{1}{2}O_{2}\to {\mathrm{CH}}_{3}\mathrm{OH}\;\Delta H_{298K}=-128\;\mathrm{kJ}\;{\mathrm{mol}}^{-1} \end{array} \end{array}$$

In conclusion, these CH_4_ reforming processes have some limitations. The CDM and DRM processes have a fatal problem: the deactivation of catalysts is caused by carbon deposition.^[^ ^8^ ^]^ Unlike Ni‐based catalysts, noble metal (e.g., Pt, Ru, Rh) catalysts are generally more resistant to deactivation due to coking, but the high cost limits the wide application of noble metal‐based catalysts in methane reforming reactions.^[^ ^35^, ^64^ ^]^ The SMR process must be carried out at a high temperature; therefore, an external heating source is required, and the per‐power conversion rate is low. In addition, the reforming reaction requires an adjustment to the H_2_/CO ratio, and the syngas ratio produced by SMR is higher than 2, which is not conducive to FTS.^[^ ^16^ ^]^ The reaction speed of the POM process is faster than that of SMR. It is an autothermal process that can be operated under high‐space‐velocity conditions without an external heating source. However, POM has strict requirements for reactor materials and troublesome operation steps that limit its industrial application. Consequently, interest in alternative reforming processes (nonconventional) has increased, and PARM is considered to have great potential in this area.^[^ ^17^, ^111^ ^]^ The need to optimize the conventional CH_4_ reforming process has led to the rapid development of PARM.

### 2.2 The Concept of Plasma‐Assisted Reforming

Plasma techniques have great potential in chemical synthesis, material modification, pollution control, medical research, and energy storage, among others.^[^ ^112^, ^113^, ^114^, ^115^, ^116^ ^]^ For the use of reforming CH_4_, the bulk temperature of the plasma reaction system can be adjusted to between room temperature and several hundred degrees Celsius by controlling the discharge parameters and external heating or cooling. This is predominant why plasma technology has attracted considerable attention in the field of CH_4_ reforming. Several PARM methods have been developed for this purpose. However, PARM is a highly sophisticated chemical reaction process in which the discharge parameters can affect the conversion rates and product species through the excitation and ionization of a neutral species via electron impact and electron‐ion dissociation and recombination.^[^ ^117^, ^118^, ^119^ ^]^ Typical discharge parameters include frequency, pressure, voltage, input power, rise time, and pulse time,^[^ ^120^ ^]^ the main mechanism of which is the interaction of plasma with the CH_4_ molecules to produce CH*_x_* radicals to induce elementary plasma‐chemical reactions (scattering, excitation, attachment, ionization, and dissociation). The characteristics of plasma provide more possibilities for methane reforming technology. Compared with the conventional methane reforming process, PARM usually does not require an additional heating process, therefore, it can effectively avoid the greenhouse gas (CO_2_) generated by the combustion of fossil fuels during the heating process.^[^ ^19^, ^23^, ^31^, ^40^, ^121^ ^]^ However, there is still a gap between PARM and conventional methane reforming processes in terms of operating efficiency and stability.

**Figure 3:**
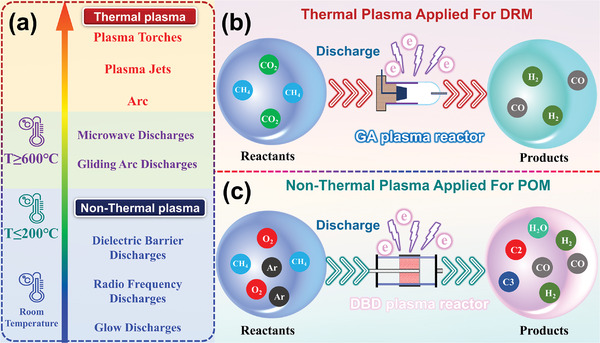
a) Temperature characteristics of several types of plasma applied in the PARM system.^[^ ^18^, ^126^ ^]^ b) Thermal plasma applied for DRM. Adapted with permission.^[^ ^127^ ^]^ Copyright 2019, Elsevier. c) Nonthermal plasma applied for POM. Reproduced with permission.^[^ ^128^ ^]^ Copyright 2019, Elsevier.

In addition, PARM can be operated at a wide range of temperatures. In general, the PARM systems are divided into thermal plasma and nonthermal plasma systems according to the average temperature of the plasma,^[^ ^31^ ^]^ as shown in **Figure** **3**. In a nonthermal plasma system, it is generally believed that the plasma phase does not reach an equilibrium state, and free radicals and excited‐state particles are formed through the collision reaction of electrons and gas molecules. The final products are obtained through these highly active components' propagation and recombination reactions. Furthermore, in nonthermal plasmas, the number and density of electrons and positive ions are usually equal or similar. Nonetheless, negative ions with high electron affinity are also efficiently formed in “electronegative” gases (such as O_2_).^[^ ^97^ ^]^ During a typical plasma chemical reaction, electrons gain energy from an electric field and are converted into energetic electrons. The energetic electrons then transfer energy to all the other plasma components through collisions, providing energy for processes such as the ionization, excitation, dissociation, attachment, and scattering of these components. However, the electron collision ionization threshold energy is too high and it is usually difficult to induce secondary electrons. Therefore, electron collision excitation and dissociation play critical roles in the PARM reaction. Unlike a nonthermal plasma system, a thermal plasma system can directly dissociate or ionize the reaction gas in the thermal equilibrium state by utilizing high temperatures in the plasma zone.^[^ ^66^ ^]^ In addition to high temperatures, thermal plasma is also characterized by high enthalpy, energy density, and chemical reactivity. Therefore, the collision rate of various particles in thermal plasma is high.^[^ ^122^ ^]^ Compared with CH_4_, these diluent gases have lower breakdown voltages, thus improving the electron energy distribution function (EEDF) in thermal plasmas and changing the discharge characteristics and reaction paths. The addition of diluent gases enhances the ionization and dissociation efficiencies of the reactant molecules during methane reforming. Heat transfer in the methane reforming process occurs between the thermal plasma and reactant molecules, which is similar to the conventional CH_4_ reforming process. Therefore, thermal decomposition plays a vital role in thermal plasma‐assisted CH_4_ reforming.

The PARM system needs to overcome the high stability of reactant molecules during operation (CH_4_, CO_2_, H_2_O, etc.), the decomposition or activation of the reactant molecules is a thermodynamically unfavorable process because a large amount of energy is required to break the chemical bonds.^[^ ^123^ ^]^ From this point of view, the thermal plasma system seems to be more suitable for the methane reforming process because thermal plasma can provide higher energy density and temperature. The literature also points out that thermal plasma‐assisted methane reforming systems have better treatment capacity and energy conversion efficiency than nonthermal plasma systems.^[^ ^124^ ^]^ However, severe quenching requirements and electrode problems limit the applicability of thermal plasmas.^[^ ^125^ ^]^ Compared with thermal plasma, nonthermal plasma has a lower degree of ionization, resulting in a lower average temperature inside the reactor.^[^ ^55^ ^]^ This enables the methane reforming process to be carried out under relatively mild conditions. Furthermore, in the thermal plasma‐assisted methane reforming system, continuous operation is enabled since ions, atoms and molecules remain at relatively low temperatures and do not etch the surfaces of the electrodes and catalysts they contact. Although theoretically, the non‐thermal plasma system is suitable for the POM process since POM is an exothermic process (*∆H*_298 k_ = ‐36 kJ mol^−1^), while thermal plasma systems show advantages in methane reforming processes requiring high temperature (DRM, MD, and SMR). However, the non‐thermal plasma system has attracted more attention in all PARM routes for the comprehensive consideration of environmental protection, economy, and stability. Most published research on PARM has been based on nonthermal plasmas.^[^ ^124^ ^]^

### 2.3 Representative Reactors Used in PARM

In PARM, many types of reactors that can produce thermal or non‐thermal plasma have been systematically studied, including DBD, MW, GAD, PTP, RF, and spark discharges. Their structural design, plasma temperature, plasma generation mode, discharge power, electric field value, power supply configuration, operating pressure, and energy density differ. Therefore, different CH_4_ reforming routes vary by reactor type.^[^ ^129^, ^130^, ^131^ ^]^ Many patents have been produced for the reactor design of the PARM system.^[^ ^132^, ^133^, ^134^ ^]^ **Table** **1** summarizes the typical structures and characteristics of several representative PARM reactors.

**Table 1:** Typical schematic view and characteristics of representative reactor applied in the CH_4_ reforming

Reactor type	Typical schematic view	Typical characteristics	Refs.
Coaxial DBD reactor	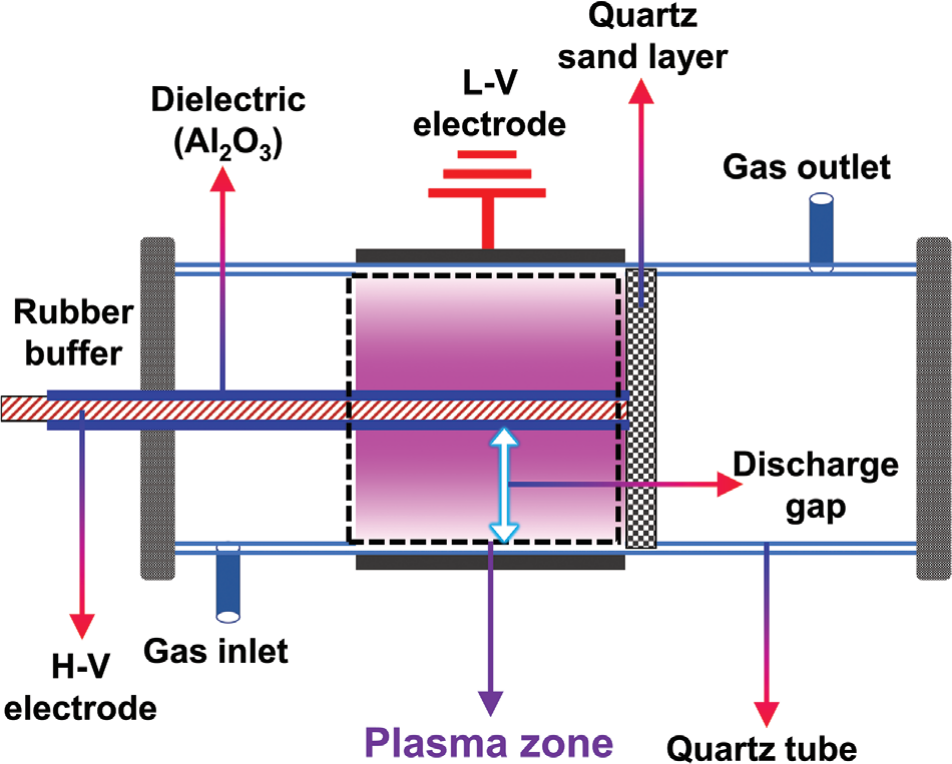	The formation of plasma is achieved by applying a high electric field between two electrodes. Simplicity, low cost, and low operating pressure and temperature; discharge is uniform, and the plasma generation process is stable. The catalyst can be placed directly in the discharge area.	[135, 136, 137, 138, 139, 140, 141, 142, 143, 144, 145]
Parallel‐plate DBD reactor	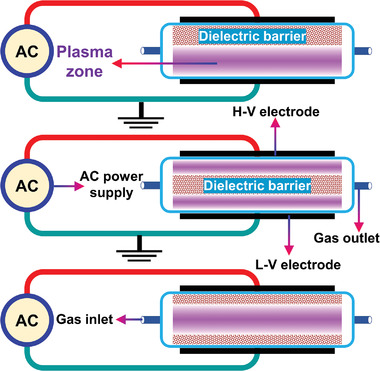	Typical characteristics are similar to coaxial DBD reactors. Poor air tightness and increased operating costs due to its parallel‐plate structure.	[19, 29, 146, 147]
MW discharge reactor	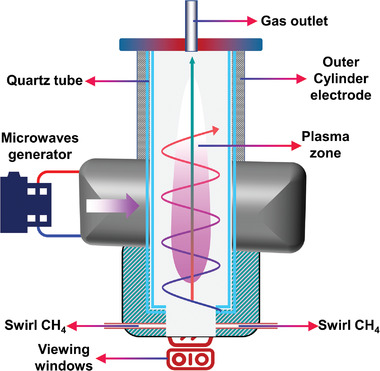	The reactor has no internal electrodes, only the reactants in the plasma area; it can provide higher energy density and reaction temperature than other reactors. Plasma can be confined in a specific space by a magnetic field. Quiet in most situations, can stably produce plasma at high pressure. High input power.	[73, 148, 149, 150, 151, 152, 153]
GAD reactor	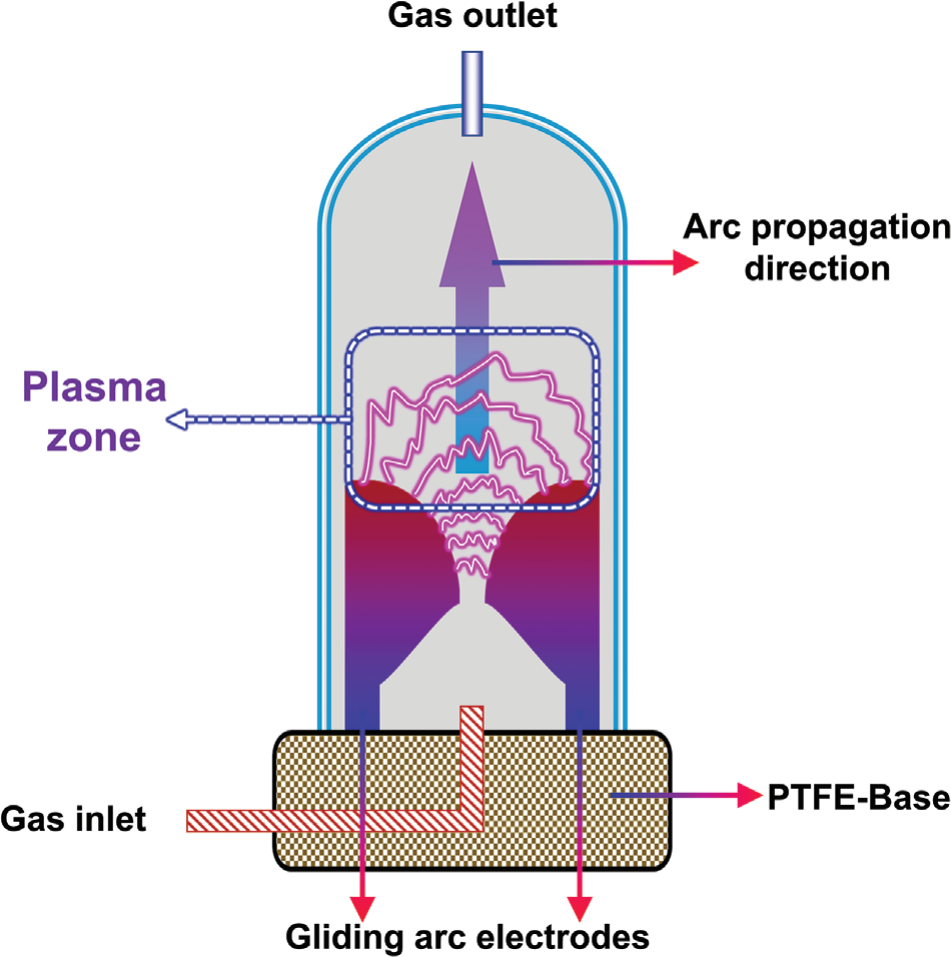	Compact structure, fast start‐up, and response, good chemical selectivity, high power density, and low energy consumption. Thermal plasma is generated by the discharge process.	[28, 154, 155, 156, 157, 158]
RF discharge reactor	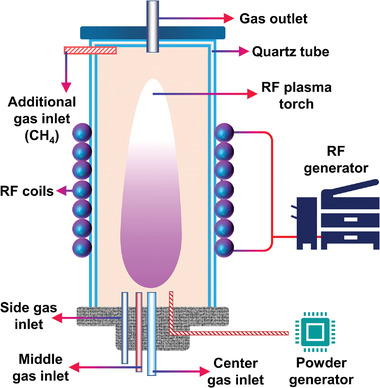	RF plasma torches are stable and have a controlled heating path. The electrode is located outside the plasma reactor, which can avoid the corrosion and pollution of the electrode by the plasma; Can generate exceptionally high‐temperature (≈8000 K/7727 °C) fields and fast quenching rates (≈106 K s^−1^).	[151, 159, 160, 161, 162, 163, 164]
PTP discharge reactor	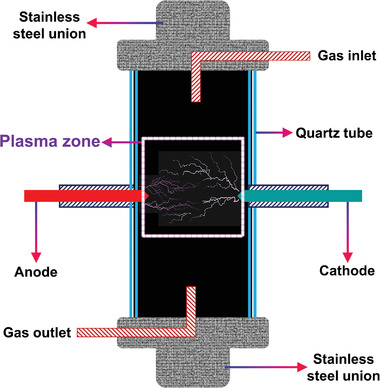	The gas temperature downstream of the plasma space is higher than that upstream due to uneven discharge in the reactor interior space, and the space of the reactor cannot be used effectively. The high temperatures will lead to carbon deposition on the electrode surface, and the discharge cannot be carried out normally.	[129, 165]

#### 2.3.1 DBD

DBD was first reported by Siemens in 1857.^[^ ^166^, ^167^ ^]^ A typical DBD reactor usually consists of two electrodes with an insulating medium inserted between them. Insulating media are usually composed of materials with low dielectric loss and high breakdown strength, such as glass, quartz, ceramics, and polymers.^[^ ^125^, ^168^ ^]^ The purpose of placing an insulating medium in the discharge gap is to limit the current flow and prevent the formation of sparks and arcs. A sinusoidal AC high‐voltage power supply typically drives a DBD. With an increase in the supply voltage, the reactive gas in the system gradually changes from the insulating state to the discharge state and finally breaks down. When the supplied voltage is relatively low, it is insufficient to cause a plasma reaction of the gas in the reaction area. As the supply voltage gradually increases, the number of electrons in the reaction region also increases. However, when the breakdown voltage of the reactive gas is not reached, the electric field between the two electrodes is relatively weak, which cannot provide sufficient energy for the electrons to collide with the gas molecules inelastically to generate the “electron avalanche” effect. Therefore, the reaction gas is still insulating, and discharge cannot be generated. Suppose the supply voltage continues to increase when the electric field between the two electrodes is strong enough to cause inelastic collisions between the electrons and gas molecules. In that case, the gas breaks down, and a discharge occurs. When the electron density in the space is higher than the critical value, the number of microdischarge channels will be generated between the two electrodes, luminescence can be observed in the reactor, and the current increases rapidly with the increase in the applied voltage. The dissociation of gas molecules occurs primarily in the microdischarge channel (plasma region) between the two electrodes. The electron temperature in the DBD reactor is usually between 1 and 30 eV, while the reaction gas temperature is usually only 30–300 °C; hence, the plasma generated by the DBD reactor is a typical non‐thermal plasma.^[^ ^27^, ^169^ ^]^ There are two mechanisms for dissociating gas molecules in the microdischarge channel. For the first mechanism, the electrons obtain energy from a strong electric field and then collide with gas molecules to generate more electrons, resulting in electron avalanches. Some atoms or molecules are excited, and active particles, such as ions and free radicals, are generated. For the second mechanism, some of the excited electrons have higher energy, which can excite gas molecules through inelastic collisions. Overall, this mechanism makes it easy to obtain free radicals, ions, excited atoms, and molecules in the plasma. The DBD technology can generate a homogenized bulk volume of plasma with a stable plasma area to ensure a uniform gas treatment. In addition, DBD technology has easy upscaling opportunities, low energy consumption, and mild operating conditions compared with other plasma technologies.^[^ ^115^ ^]^ Although many different types of plasma reactors have been studied for PARM applications, DBD has been the most frequently studied.^[^ ^170^, ^171^ ^]^ Nevertheless, solving the low energy efficiency of DBD plasma when applied to CH_4_ reforming is a considerable challenge.

#### 2.3.2 MW

MW has wavelengths between 1 and 1000 mm, corresponding to frequencies from 0.3 to 300 GHz.^[^ ^172^ ^]^ The most common frequencies are 900 MHz and 2.45 GHz.^[^ ^173^ ^]^ The frequency of 2.45 GHz is most commonly used in all household microwave ovens and laboratory‐scale microwave reaction systems.^[^ ^174^ ^]^ In 1967, MW was first used in chemical research.^[^ ^175^ ^]^ Since then, microwave technology has been widely used in many fields, such as the chemical industry, materials, medicine, biochemistry, and catalysis.^[^ ^176^ ^]^ In recent years, the application of MW has resurged in CH_4_ reforming reactions. MW plasma is considered to have the highest CH_4_ conversion and energy efficiency.^[^ ^152^ ^]^ Its parameters can vary over a wide range depending on the operating conditions, and its plasma types can range from nonthermal to near thermal.^[^ ^177^ ^]^ Compared with conventional CH_4_ reforming technologies, the main attractiveness of MW technology is its shorter reaction time,^[^ ^178^, ^179^ ^]^ and the feature of automatic operation provides better safety and environmental compatibility.^[^ ^180^, ^181^ ^]^ The short reaction times achieved by MW can effectively improve the energy utilization efficiency, especially for large‐scale applications in multimode MW reactors.^[^ ^148^ ^]^ In addition, MW can also maintain the stability of the catalyst in the CH_4_ reforming reaction because it can effectively avoid carbon deposition on the active center of the catalyst,^[^ ^182^, ^183^ ^]^ which is in contrast to the conventional CH_4_ reforming reaction. However, the disadvantage of MW‐based CH_4_ reforming technology is the high input power required to maintain stable plasma conditions. The combination of a catalyst and MW plasma enables the use of a reduced reactor size, enabling the development of a lightweight CH_4_ reforming reactor, which is well suited for medium‐sized plants. Despite the promising applications of MW plasma in the field of CH_4_ reforming, the amount of research focusing on this topic is limited.

#### 2.3.3 GAD

The plasma generated by the arc is divided into two types: transferred arc and non‐transferred arc.^[^ ^184^ ^]^ In a transferred arc reactor, a gas or catalyst can act as one of the electrodes and be directly exposed to the arc plasma. The temperature of the arc plasma region is usually between 4500 to 10000 °C.^[^ ^27^, ^185^ ^]^ Three parameters, electron density, Joule heating, and pressure can significantly affect the temperature of the arc plasma.^[^ ^32^, ^185^, ^186^ ^]^ Notable, the heat transfer in the arc thermal plasma‐assisted reforming of the CH_4_ process is similar to the conventional thermal process.^[^ ^184^ ^]^ In nontransferred arc reactors, the gas is typically treated using the plasma jet, leaving the torch that holds the arc. The temperature of these plasma jets is between 10 000 and 20 000 °C, depending on the plasma gas, power level, and design of the torch.^[^ ^187^ ^]^ For PARM, a transferred arc reactor is preferable. As the most studied arc reactor in the field of CH_4_ reforming, the GAD reactor has the advantages of compact design, high CH_4_ conversion, good chemical selectivity, high energy density, high energy efficiency ratio, and low energy consumption.^[^ ^188^ ^]^ The arc generated during the discharge of a 2D device has defects such as breakdown, extinction, and re‐breakdown, resulting in an inhomogeneous distribution of active species and plasma, and its conversion rate for MD, DRM, and POM reactions is only 4 to 65%.^[^ ^81^, ^189^, ^190^, ^191^ ^]^ Rotating sliding arc (RGA) discharge can overcome the above problems to some extent by periodically rotating the arc. In most reports, RGA plasma is defined as thermal plasma, which can improve the efficiency of the electrochemical reactions because thermal plasma has a higher electron density than nonthermal plasma sources.^[^ ^155^ ^]^ This means that the RGA plasma has relatively high electron energy and moderate gas temperature, which is beneficial for improving the CH_4_ conversion rate.^[^ ^154^ ^]^ Arcs in RGA reactors can be generated by applying a discharge to a pair of electrodes in the laminar or turbulent gas flow, and a complete discharge cycle consists of three main stages: breakdown, balanced arc, and unbalanced arc.^[^ ^192^, ^193^ ^]^ In 3D space, when the applied voltage reaches the critical value of gas breakdown, the arc is always generated from the closest distance to the electrode. The arc length is extended due to the arc sliding caused by the rotating gas flow. During the arc lengthening process, the electrical properties of the arc constantly change until the arc is no longer self‐sustaining, and then a new cycle begins.^[^ ^155^, ^194^, ^195^, ^196^ ^]^ The RGA reactor system is not complicated, and its unique discharge phenomenon meets the requirements of high productivity and good adaptability of the PARM, showing great potential for commercialization.

#### 2.3.4 RF

In a system that generates RF plasma, electromagnetic energy is transferred to the RF thermal plasma by inducing an RF power source. The generated RF plasma torch is fine‐tuned using an internal water‐cooled quartz tube.^[^ ^21^ ^]^ RF reactors have several advantages that make them suitable for CH_4_ reforming. First, the RF reactor allows non‐thermal plasma generation at low pressures (i.e., 1–103 Pa), which helps drive a controlled CH_4_ reforming reaction.^[^ ^163^ ^]^ Second, the RF plasma torch can be generated in the low‐frequency range of 1–100 MHz, and the volume of the generated plasma torch is equivalent to that of the entire plasma reactor, which ensures that the CH_4_ reforming process is driven with a relatively uniform plasma bulk. RF reactors are gaining industrial attention owing to their improved scalability. Third, the energy efficiency of RF reactors can be optimized using an impedance‐matching network. The electrode generating the RF plasma torch is located outside the reactor. It can avoid the electrode participating in the CH_4_ reforming reaction, which is beneficial to the PARM process.^[^ ^197^, ^198^, ^199^, ^200^ ^]^ Nevertheless, the relative abundance of the product species strongly depends on the discharge parameters of the RF reactor. The increase in the energy deposition in RF plasma torches leads to the formation of substantially more types of hydrocarbons, which is an urgent problem.

#### 2.3.5 PTP

Because of the electric field enhancement effect at the tip of sharp objects, sharp bare metals are often used as electrodes to generate various discharges.^[^ ^201^ ^]^ Accordingly, PTP reactors have been designed and used for CH_4_ reforming. Generally, PTP reactors can generate high‐frequency (8 kHz) pulsed non‐thermal plasma.^[^ ^129^ ^]^ Pulsed plasma consists of pulsed corona discharge and pulsed spark discharge, in which CH_4_ is ionized and converted. The PTP reactor comprises a Pyrex tube and two stainless steel electrodes with sharp terminals. These terminals have a small radius of curvature and lead to the formation of a particularly high electric field intensity. Finally, plasma is formed by electron emission and ionization.^[^ ^202^, ^203^ ^]^ A feed gas of CH_4_ is introduced into the upper part of the vertically placed reactor at a certain rate. C_2_ hydrocarbons are in the effluent from the lower part of the reactor. All the experiments were carried out at atmospheric pressure without external heating.^[^ ^129^ ^]^ The advantage of the PTP reactor is that it has a simple structure and can be carried out under atmospheric pressure without external heating. However, the plasma's CH_4_ conversion, product selectivity, stability, and uniformity are not satisfactory. As a result, there are few relevant studies.

## 3 Application of Plasma Technology for Different CH_4_ Reforming Routes

Similar to the conventional methane reforming process, the PARM process can be divided into four paths according to the difference in the feedstock:^[^ ^8^, ^36^, ^40^ ^]^ plasma‐assisted methane decomposition (PAMD, feedstock: CH_4_ or CH_4_+inert gas), plasma‐assisted dry reforming of methane (PADRM, feedstock: CH_4_+CO_2_ or CH_4_+CO_2_+inert gas), plasma‐assisted partial oxidation of methane (PAPOM, CH_4_+O_2_), and plasma‐assisted steam methane reforming (PASMR, feedstock: CH_4_+water vapor). Currently, there has been a large amount of research on PARM, and the experiments have reached a high degree of sophistication. While DBD systems still dominate PARM‐related research, the range of plasma reactors used is increasing. Presently, the vast majority of PARM system research is laboratory‐scale, although RGA and MW plasma systems offer higher throughput for a single reactor, while other designs will require multiple reactors in parallel to achieve this. PARM is a very sophisticated system in which the conversion of reactants and product distribution (selectivity) are affected by the catalyst (metal type, structure, and position in the reactor), plasma reactor (type, size, material, and structure), feed (ratio and flow rate), and discharges parameter (voltage, frequency, energy density, etc.). This makes it difficult to compare the pros and cons of different studies in detail. In the following content, we systematically summarize and analyze the four PARM paths, especially the basic elementary plasma‐chemical reaction processes of different reactant molecules in the PARM systems.

### 3.1 PAMD

**Figure 4:**
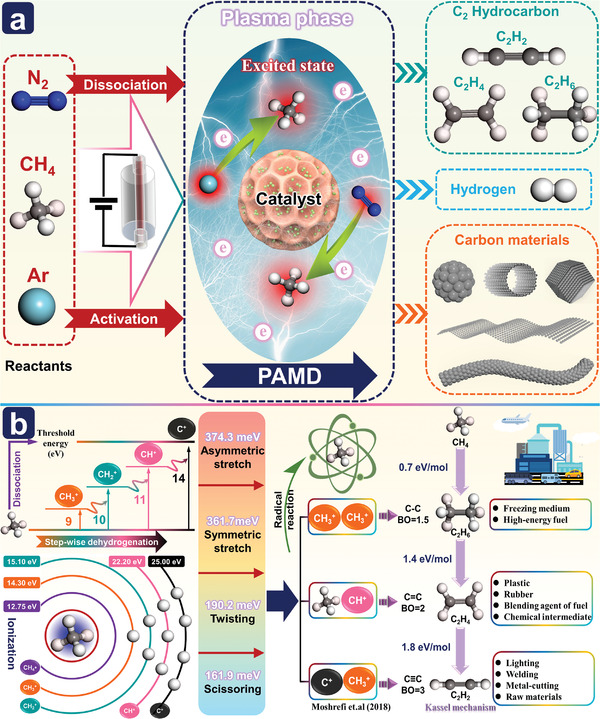
a) Schematic diagram of possible plasma‐assisted methane decomposition process; b) Possible elementary plasma‐chemical reactions in PAMD process.

Because of the exceptionally strong C—H bonds, thermal (noncatalytic) decomposition of CH_4_ takes place at extremely high temperatures (>1200 °C). This is an endothermic reaction; at sufficiently high temperatures, CH_4_ can be decomposed into H_2_ and solid carbon, and a series of higher hydrocarbons can also be synthesized; namely, C_2_H_6_, C_2_H_4_, C_2_H_2_, etc.^[^ ^38^ ^]^ Plasma‐assisted methane decomposition (PAMD) system can initiate a chemical reaction at a lower operating temperature via high‐energy electron collisions with the CH_4_ molecules.^[^ ^158^ ^]^ In a plasma reactor, free electrons can be accelerated by strong electric or magnetic fields, thereby increasing their kinetic energy. Generally, in the plasma bulk, the temperature of electrons ranges from 1 to 5 eV. These high‐energy electrons collide with the CH_4_ molecules to provide energy for the plasma chemical reaction of the active species. The rates of these fundamental reactions depend on the number of effective collisions of the active species. Usually, low‐energy electrons can lead to vibrational excitation, medium‐energy electrons to molecular dissociation, and high‐energy electrons to molecular dissociation.^[^ ^177^ ^]^ In the PAMD reaction, because the CH_4_ molecule contains five atoms and four C—H bonds, its vibrational mode, and dissociation process are complex, and the energy required to break the four C—H bonds is high. Accordingly, it is difficult to directly dissociate CH_4_ through electron collisions. Possible electron collision reactions are shown in **Figure** **4**b and R3.1–R3.13. The threshold energies for the dissociation reactions R3.1–R3.4 are 9, 10, 11, and 14 eV, respectively.^[^ ^31^ ^]^ These threshold energies are high for conventional plasmas. Nonetheless, the dissociation of CH_4_ molecules can occur via collisions between free electrons and excited CH_4_. Additionally, Morgan^[^ ^204^ ^]^ demonstrated that CH_4_ dissociation can be induced by two vibrational channels, including reactions R3.5–R3.8, where the threshold energies of reactions R3.5 and R3.6 are 0.162 eV and 0.361 eV, respectively, which are easier to induce than electron collision dissociation. However, the formation of H_2_ from H atoms is an exothermic reaction, which usually occurs in the form of a three‐body collision. Therefore, regardless of the type of discharge or reactor, the gas temperature will inevitably increase during the discharge process.

Dissociation:

(R3.1) $$e+{\mathrm{CH}}_{4}\to e+{\mathrm{CH}}_{3}+H\;\mathrm{Threshold}\;\mathrm{energy}=9\;\mathrm{eV}$$

(R3.2) $$e+{\mathrm{CH}}_{4}\to e+{\mathrm{CH}}_{2}+H_{2}\;\mathrm{Threshold}\;\mathrm{energy}=10\;\mathrm{eV}$$

(R3.3) $$e+{\mathrm{CH}}_{4}\to e+H_{2}+\mathrm{CH}+H\;\mathrm{Threshold}\;\mathrm{energy}=11\;\mathrm{eV}$$

(R3.4) $$e+{\mathrm{CH}}_{4}\to e+C+2H_{2}\;\mathrm{Threshold}\;\mathrm{energy}=14\;\mathrm{eV}$$

Vibrational excitation:

(R3.5) $$e+{\mathrm{CH}}_{4}\to e+{\mathrm{CH}}_{4}\left(v24\right)\;\mathrm{Threshold}\;\mathrm{energy}=0.162\;\mathrm{eV}$$

(R3.6) $$e+{\mathrm{CH}}_{4}\to e+{\mathrm{CH}}_{4}\left(v13\right)\;\mathrm{Threshold}\;\mathrm{energy}=0.361\;\mathrm{eV}$$

(R3.7) $$e+{\mathrm{CH}}_{4}\left(v24\right)\to e+{\mathrm{CH}}_{3}+H$$

(R3.8) $$e+{\mathrm{CH}}_{4}\left(v13\right)\to e+{\mathrm{CH}}_{3}+H$$

Ionization:

(R3.9) $$e+{\mathrm{CH}}_{4}\to {\mathrm{CH}}_{4}^{+}+2e\;\mathrm{Threshold}\;\mathrm{energy}=12.75\;\mathrm{eV}$$

(R3.10) $$e+{\mathrm{CH}}_{4}\to {\mathrm{CH}}_{3}^{+}+H+2e\;\mathrm{Threshold}\;\mathrm{energy}=14.3\;\mathrm{eV}$$

(R3.11) $$e+{\mathrm{CH}}_{4}\to {\mathrm{CH}}_{2}^{+}+H_{2}+2e\;\mathrm{Threshold}\;\mathrm{energy}=15.1\;\mathrm{eV}$$

(R3.12) $$\begin{array}{c} e+{\mathrm{CH}}_{4}\to {\mathrm{CH}}^{+}+H_{2}+H+2e\;\mathrm{Threshold}\;\mathrm{energy}=22.2\;\mathrm{eV} \end{array}$$

(R3.13) $$e+{\mathrm{CH}}_{4}\to C+2H_{2}+2e\;\mathrm{Threshold}\;\mathrm{energy}=25\;\mathrm{eV}$$

The basic elementary plasma‐chemical reaction process of CH_4_ molecules is different in thermal and nonthermal plasma systems. In the nonplasma reactor, electron energy follows the Maxwell Boltzmann distribution and the temperature of the electrons ranges between 1 and 5 eV (these values are lower than the threshold energies (9 eV) for the direct dissociation of CH_4_ molecules) in the reactor.^[^ ^177^ ^]^ This also causes CH_4_ scattering and a uniform density distribution is finally achieved. Although the direct dissociation of CH_4_ molecules is difficult, it is relatively easy to dissociate CH_4_ molecules in the excited state. As shown in Figure 4b, CH_4_ molecules can be impacted with low‐energy electrons and excited into four fundamental modes, namely, CH_4_ (*ν*_1_, symmetric stretch), CH_4_ (*ν*_2_, twisting), CH_4_ (*ν*_3_, asymmetric stretch), and CH_4_ (*ν*_4_, scissoring).^[^ ^205^ ^]^ Furthermore, the threshold energies for the above four modes are 361.7 meV, 190.2 meV, 374.3 meV, and 161.9 meV, respectively. These excited states of CH_4_ have higher internal molecular energies; as a result, the threshold energy required for electron impact dissociation can be reduced.^[^ ^204^ ^]^ In the thermal plasma dissociation of CH_4_, a large number of excited singlet states CH_4_ (S_1_) (9.6 eV) and CH_4_ (S_2_) (11.7 eV) are formed owing to the electron‐molecule interactions.^[^ ^206^ ^]^ These excited singlet state CH_4_ molecules are extremely unstable and can be rapidly decomposed into CH_3_, CH_2_, CH, C, and other free radicals.^[^ ^207^, ^208^ ^]^ In thermal plasmas, CH_4_ molecules have three excitation modes, namely, rotational, vibrational, and electronic, which correspond to typical threshold energies of 0.01 to 0.1, 0.1 to 1, and 1 to 10 eV, respectively. Lee et al.^[^ ^209^ ^]^ found that the dissociation of CH_4_ by electron collisions requires high electron energies above 9 eV, and CH_4_ molecules can be directly ionized if the electron energy exceeds 12.75 eV.^[^ ^210^, ^211^ ^]^ Some studies have demonstrated that the ionization and dissociation efficiency of CH_4_ can be effectively enhanced by the addition of noble gases during thermal plasma‐assisted CH_4_ reforming and that these gases can be excited to metastable levels and ionized states while more efficiently improving the EEDF values in the gas discharge state.^[^ ^19^, ^212^ ^]^

As shown in Figure 4a, the products of PAMD mainly include H_2_, carbon products, and C_2_ hydrocarbons, and the selectivity of the PAMD system for these products can be flexibly adjusted according to different needs.

#### 3.1.1 Carbon Products Production Via PAMD System

Pristavita et al.^[^ ^22^ ^]^ used an inductively coupled thermal plasma torch system of 35 kW to generate carbon black nanopowders. They found that the morphology of the products was affected by the experimental pressure. Lower pressure means generating larger temperature gradients (i.e., carbon particles tend to form amorphous structures in high‐temperature regions). In contrast, crystalline powders were obtained at higher pressures related to higher temperatures. The plasma‐assisted decomposition of CH_4_ could obtain carbon nanopowders with a crystalline graphitic structure, and these carbon nanopowders were uniformly distributed in the reactor.^[^ ^213^ ^]^ Furthermore, the addition of nitrogen did not affect the morphology of the powders while promoting the formation of a pyridinic type of N‐bonding. In addition, Pristavita et al.^[^ ^214^ ^]^ simulated the entire process control for reproducibility and uniformity and obtained the desired structures for carbon black produced in the thermal plasma‐assisted decomposition of CH_4_. Further numerical simulations have been performed on the flow, energy field, flow function line, and quenching rate of the plasma gas in the reactor under different pressures. The results indicate that in the pressure range of 20.7–101.3 kPa and plasma power range of 10–20 kW, stable process control can cause the obtained carbon black particle morphology to change very little, and the particle morphology has exceptionally high reproducibility. Baldissarelli et al.^[^ ^215^ ^]^ used a thermal plasma direct current (DC) system through the plasma pyrolysis of CH_4_ to form carbon black and carbon nanotubes. The results demonstrate that carbon nanotubes were produced in the presence of catalysts (Ni‐ and Ce‐Al_2_O_3_), while carbon black formation occurred without catalysts. This indicates that atomic carbon diffusion at the surface of the heated catalyst promotes its precipitation into the graphene sheets. As carbon nanotubes grow on the catalyst surface, the active metal component of the catalyst is pushed upward, forming a metal cap. The catalyst is deactivated when the active metal component is completely solidified and encapsulated by the graphene layer, and the nanotube growth stops. As for carbon black, Gautier anticipated that gas‐phase carbon particle nucleation and growth are vital during the entire process.^[^ ^216^ ^]^ Li et al.^[^ ^217^ ^]^ A 2 kW DC thermal arc plasma setup was used in an Ar atmosphere to generate the products. The experimental results suggest that the formation of solid carbon during plasma‐assisted decomposition of CH_4_ was determined by the feed gas's residence time and feed flow rate and amount of quench gas. They also found that the formation of methyl and C_2_ radicals is a precursor of solid carbon.

#### 3.1.2 C_2_ Hydrocarbon Products Production via PAMD System

Zhang et al.^[^ ^188^ ^]^ used a GAD reactor for CH_4_ + N_2_ plasma treatment to generate C_2_ hydrocarbons. They found that N_2_ could promote the dissociation of CH_4_. The formation of C_2_ hydrocarbons is affected by CN and C_2_ radicals. The CN radicals in the plasma phase originate from the reaction between the N_2_ molecules or N and CH*_x_* radicals, while the C_2_ radicals originate from the reaction of atomic C with C‐containing species, such as atomic C, CN, and CH radicals. In the reactor of this study, the CN vibration temperature and C_2_ rotation temperature were 0.56–0.86 eV and 1052–1713 °C, respectively. Yao et al^[^ ^129^ ^]^ PTP and CAC plasmas were used to determine the formation mechanism of C_2_ hydrocarbons as follows: CH_4_ → C_2_H_6_ → C_2_H_4_ →C_2_H_2_. They also proposed a higher gas flow rate and lower residence time for the PTP reactor to improve yield. This mechanism agrees with that of Wnukowski et al.^[^ ^152^ ^]^ they studied the effect of H_2_ addition on CH_4_ coupling in an MW moderate‐pressure plasma reactor. The results suggest that adding H_2_ to the inlet gas can improve the conversion of CH_4_ and the yield and selectivity of acetylene and ethylene. The addition of H_2_ provides an additional pool of H radicals that play a vital role in the decomposition of CH_4_. Moreover, the presence of H radicals enhances the formation of C_2_H_2_ and C_2_H_4_. Additional H_2_ can consume the C_2_H radicals (initial soot precursors) to generate C_2_H_2_. Some researchers have proposed that DBD plasma can generate C_2_ hydrocarbons and H_2_.^[^ ^218^, ^219^, ^220^ ^]^ Aghamir et al.^[^ ^143^ ^]^ found that DBD plasma had a relatively low conversion of CH_4_ to generate products. Xu et al.^[^ ^220^ ^]^ investigated the relationship between the electron energy distribution function (EEDF) and the H radical concentration in the PAMD process using 1D particle‐in‐cell Monte Carlo collision and fluid dynamic models. The results indicate that the formation and development process of the streamer is opposite to the evolution characteristics of the EEDF; when the applied voltage increases, the proportion of electrons with high energy increases, which promotes the ionization and dissociation of CH_4_ and increases the H density.

The C_2_ hydrocarbons acetylene (C_2_H_2_), ethylene (C_2_H_4_), and ethane (C_2_H_6_) are high‐value feedstocks for the chemical industry.^[^ ^221^, ^222^, ^223^, ^224^ ^]^ The direct plasma conversion of CH_4_ to C_2_ hydrocarbons is illustrated in reaction R3.14–R3.16, a strongly endothermic reaction,^[^ ^151^ ^]^ and relatively large amounts of energy are required per unit mass of C_2_ hydrocarbons formed.

(R3.14) $$2{\mathrm{CH}}_{4}\to C_{2}H_{2}+3H_{2}\;\Delta H_{298K}=376.47\;\mathrm{kJ}\;{\mathrm{mol}}^{-1}$$

(R3.15) $$2{\mathrm{CH}}_{4}\to C_{2}H_{4}+2H_{2}\;\Delta H_{298K}=202.21\;\mathrm{kJ}\;{\mathrm{mol}}^{-1}$$

(R3.16) $$2{\mathrm{CH}}_{4}\to C_{2}H_{6}+H_{2}\;\Delta H_{298K}=220.88\;\mathrm{kJ}\;{\mathrm{mol}}^{-1}$$

CH_4_ has a low dissociation energy (4.45 eV), and the threshold energy of direct dissociation of CH_4_ is 9 eV.^[^ ^19^, ^204^ ^]^ Hence, it can be dissociated in plasma treatment (non‐thermal plasma with threshold energy between 1 and 10 eV and thermal plasma with more than 10 eV). The possible reaction mechanisms and forward rate coefficients of C_2_ hydrocarbon formation are summarized in **Table** **2**. In theory, the MD process requires a high temperature (>1273 K) to induce the upper path.^[^ ^177^ ^]^ Because this is a stepwise process, the distribution of the decomposition products is usually determined by temperature and reaction time. Many studies have indicated that the primary product of CH_4_ decomposition is C_2_H_6_; however, C_2_H_6_ is rapidly further dehydrogenated to other products.^[^ ^225^ ^]^ This phenomenon is caused by the stability of the different products at high temperatures (required for a sufficient CH_4_ conversion rate).^[^ ^226^, ^227^ ^]^ Therefore, long reaction times and high decomposition temperatures favor the formation of unsaturated C_2_ compounds, and when the conditions are harsh enough, C_2_H_4_ and C_2_H_2_ are the final products. Regardless of the products of CH_4_ decomposition, the process begins with breaking the C—H bond, which has high activation energy and is the rate‐controlling step of the entire decomposition process.^[^ ^225^, ^228^, ^229^ ^]^ For the PTP reactor for the MD process, only a small part of CH_4_ is in a state of excitation when the CH_4_ molecules pass through the discharge region, leading to lower conversion of CH_4_. Subsequently, CH_4_ dissociates into CH_3_ and H radicals. Two CH_3_ radicals can dimerize to generate C_2_H_6_, and CH_3_ radicals can generate CH*_x_* via thermal dehydrogenation or H extraction mechanisms. Subsequently, C_2_ compounds can be produced through two major routes: thermal dehydrogenation of C_2_H_6_ (possibly involving an H‐extraction mechanism) and CH*_x_* radical coupling.^[^ ^153^, ^230^, ^231^ ^]^

**Table 2:** Reaction mechanism and forward rate coefficients of C_2_ hydrocarbons formation^[^ ^153^, ^206^, ^232^ ^]^

No.	Reaction	*K*_(_ *_T_*_)_ = *AT^n^* exp(−*E*/*RT*)		
		*A*	*n*	*E* [kJ mol^−1^]
1	CH_4_ +H → CH_3_ + H_2_	1.30 × 10^4^	3.0	33.6
2	CH_4_ + CH → C_2_H_4_ + H	3.00 × 10^13^	0.0	‐1.7
3	CH_4_ + CH_2_ → CH_3_ + CH_3_	1.30 × 10^13^	0.0	39.9
4	CH_3_ + H → CH_4_	1.93 × 10^36^	−7.0	38.00
5	CH_3_ + CH_3_ → C_2_H_6_	1.69 × 10^53^	−12	81.24
6	CH_3_ + CH_3_ → C_2_H_5_ + H	3.01 × 10^13^	0.0	56.54
7	CH_3_ + CH_3_ → C_2_H_4_ + H_2_	1.00 × 10^16^	0.0	134
8	CH_3_ + M → CH_2_ + H + M	1.00 × 10^16^	0.0	379
9	CH_3_ + M → CH + H_2_ + M	6.90 × 10^14^	0.0	345
10	CH_2_ + H → CH + H_2_	6.00 × 10^12^	0.0	‐7.5
11	CH_2_ + CH_2_ → C_2_H_2_ + H_2_	1.20 × 10^13^	0.0	3.4
12	CH_2_ + CH_2_ → C_2_H_2_ + H + H	1.10 × 10^14^	0.0	3.4
13	CH_2_ + CH_3_ → C_2_H_4_ + H	4.20 × 10^13^	0.0	0
14	CH_2_ + C_2_H → C_2_H_2_ + CH	1.81 × 10^13^	0.0	0
15	CH_2_ + CH_3_ → C_2_H_4_ + H	1.81 × 10^13^	0.0	0
16	C_2_H + H → C_2_H_2_	1.81 × 10^14^	0.0	0
17	C_2_H + CH_4_ → C_2_H_2_ + CH_3_	1.81 × 10^12^	0.0	2.08
18	C_2_H + C_2_H_3_ → C_2_H_2_ + C_2_H_2_	9.64 × 10^11^	0.0	0
19	C_2_H + C_2_H_5_ → C_2_H_2_ + C_2_H_4_	1.81 × 10^12^	0.0	2.08
20	C_2_H_2_ + H → C_2_H + H_2_	6.02 × 10^13^	0.0	116.4
21	C_2_H_3_ + H → C_2_H_2_ + H	4.73 × 10^40^	−8.8	194.5
22	C_2_H_3_ + H → C_2_H_2_ + H_2_	1.20 × 10^13^	0.0	0
23	C_2_H_3_ + CH_3_ → C_2_H_2_ + CH_4_	3.92 × 10^11^	0.0	0
24	C_2_H_3_ + C_2_H_3_ → C_2_H_2_ + C_2_H_4_	9.64 × 10^11^	0.0	0
25	C_2_H_4_ + CH_3_ → C_2_H_3_ + CH_4_	4.16 × 10^12^	0.0	45.56
26	C_2_H_4_ + H → C_2_H_3_ + H_2_	1.70 × 10^15^	0.0	62.9
27	C_2_H_4_ + M → C_2_H_2_ + H_2_ + M	2.50 × 10^17^	0.0	320
28	C_2_H_4_ + M → C_2_H_3_ + H + M	1.70 × 10^18^	0.0	404
29	C_2_H_5_ → C_2_H_4_ + H	1.02 × 10^43^	−9.1	224.15
30	C_2_H_5_ + C_2_H_2_→ C_2_H_6_ + C_2_H	2.70 × 10^11^	0.0	98.11
32	C_2_H_5_ + C_2_H_5_→ C_2_H_6_ + C_2_H_4_	1.39 × 10^12^	0.0	0
33	C_2_H_6_ + H→ C_2_H_5_ + H_2_	1.44 × 10^9^	1.5	31.1
34	C_2_H_6_ + CH_2_→ C_2_H_5_ + CH_3_	2.20 × 10^13^	0.0	36.3
35	C_2_H_6_ + CH_3_→ C_2_H_5_ + CH_4_	1.50 × 10^−7^	6.0	25.4

Catalyst can significantly improve the efficiency and product selectivity of PAMD systems. It has been reported that the rate of CH_4_ decomposition activity in conventional CH_4_ decomposition by the transition metals follows the order: Co, Ru, Ni, Rh>Pt, Re, Ir>Pd, Cu, W, Fe, Mo.^[^ ^233^ ^]^ However, other researchers found that nickel or Ni/Al_2_O_3_ and Fe/Al_2_O_3_ showed the highest activity.^[^ ^234^ ^]^ The most important factors affecting the metal‐catalyzed CH_4_ decomposition efficiency and carbon deposition are the particle size, dispersibility, and stability of the metal catalyst particles, which are controlled by selecting suitable supports.^[^ ^43^ ^]^ So far, Ni‐based catalysts have been widely used in PAMD systems (**Table** **3**). Ghanbari et al.^[^ ^218^ ^]^ experimentally investigated the catalytic conversion of CH_4_ to H_2_ using a nanosecond pulsed DBD plasma reactor. The results show that the presence of Ni‐K_2_O/Al_2_O_3_ catalyst greatly improves CH_4_ conversion, H_2_ production, H_2_ selectivity, and energy efficiency. Noble metal‐based catalysts usually have better activity in PAMD systems, and can effectively suppress coking and avoid rapid catalyst deactivation. Khalifeh et al.^[^ ^219^ ^]^ reported the experimental study of DBD plasma coupled Pt‐Re/Al_2_O_3_ catalyst for decomposing CH_4_ for H_2_ production. The experimental results show that this plasma‐catalytic system can achieve very high energy efficiencies and CH_4_ conversion when the discharge power is lower than 10 W and carbon deposition on catalyst and electrode surfaces can be significantly suppressed. Very recently, Hu et al.^[^ ^235^ ^]^ successfully prepared stable and highly dispersed Pd species on CeO_2_. The Pd species interacted strongly with the CeO_2_ support, effectively preventing the aggregation of Pd species during the high‐temperature catalytic experiments. The coupling of the Pd/CeO_2_ catalyst and plasma can significantly promote the CH_4_ conversion, and effectively improve the coke resistance of the catalyst. Pt‐based catalysts also exhibit excellent catalytic activity and coking resistance in PAMD systems. For example, Liu et al.^[^ ^236^ ^]^ reported the plasma‐catalytic direct nonoxidative coupling of CH_4_ into C_2_ hydrocarbons over ceria‐supported atomically dispersed Pt (Pt/CeO_2_‐SAC) catalysts in DBD plasma. The Pt/CeO_2_‐SAC coupled DBD plasma can achieve CH_4_ conversion of 39% and C_2_ selectivity of 54%, and the outstanding activity is attributed to abundant coordinatively unsaturated Pt sites in catalyst, which were more active for C—H bond cleavage. Furthermore, the isolated Pt atoms in Pt/CeO_2_‐SAC suppress coking by preventing the deep dehydrogenation of CH*_x_* intermediates and promoting the formation of C_2_ hydrocarbons. However, the high cost of noble metal‐based catalysts must be considered. Doping noble metals in Ni‐based catalysis may be an effective strategy to achieve a balance between anti‐coking, activity, and cost, which needs to be further studied.

**Table 3:** Summary of the performance of various PAMD systems

Plasma action stage	Catalyst	Reactor types	Feed composition	Maximum conversion [%]	Selectivity of products [%]	Refs.
Catalytic	Ni/75Al_2_O_3_SiO_2_	GAD	CH_4_ + Ar	34.0 (CH_4_)	≈80.0 (H_2_); ≈76.0 (C_2_H_2_)	[81]
	Ni‐K_2_O/Al_2_O_3_	DBD	CH_4_ + Ar	79.3 (CH_4_)	75 (H_2_); 90.0 (C_2_H_6_)	[218]
	Pt‐Re/Al_2_O_3_	DBD	CH_4_ + Ar	100.0 (CH_4_)	100.0 (H_2_)	[219]
	Ni/SiO_2_	DBD	CH_4_ + N_2_	87.0 (CH_4_)	53.7 (H_2_)	[237]
	Pd/CeO_2_	DBD	CH_4_	38.0 (CH_4_)	50 (H_2_); 6.0 (C_2_H_2_); 15.0 (C_2_H_4_)	[235]
	Pt/CeO_2_‐SAC	DBD	CH_4_	39.0 (CH_4_)	54.0 (C_2_)	[236]
Noncatalytic	None	MW	CH_4_	48.6 (CH_4_)	34.0 (C_2_H_6_); 19.0 (C_2_H_2_ + C_2_H_4_)	[152]
		DBD	CH_4_ + Ar	61.5 (CH_4_)	52.5 (H_2_)	[218]
		DC	CH_4_ + Ar	100.0 (CH_4_)	22.0 (H_2_), 70.0 (C_2_H_2_)	[217]
		DBD	CH_4_	25.2 (CH_4_)	33.0 (H_2_); 13.0 (C_2_H_2_+ C_2_H_4_); 33.0 (C_2_H_6_)	[220]
		Corona	CH_4_	44.6 (CH_4_)	30.1(C_2_H_2_); 1.5 (C_2_H_6_ + C_2_H_4_)	[238]
		PTP	CH_4_	23.5 (CH_4_)	85.0 (C_2_H_2_); 1.9 (C_2_H_6_); 5.4 (C_2_H_4_)	[129]
		PTP	CH_4_	19.7 (CH_4_)	84.0 (H_2_); 85.0 (C_2_H_4_)	[239]
		RGA	CH_4 +_ N_2_	91.8 (CH_4_)	31.7 (C_2_H_2_); 80.7 (H_2_)	[188]

Although various studies have been conducted regarding the role of different reactors in PAMD, comparing the different reactors and establishing their efficiency sequence is not simple. Reactors have been reported to decompose CH_4_ under various conditions, they differ in their structure, material, size, reaction conditions, and research goals. Even CH_4_ conversion as an evaluation index is unreasonable because the product value and selectivity also need to be considered. As shown in Table 3, the DBD reactor is the most popular in the research of plasma‐assisted methane decomposition. The plasma generated by the DBD reactor is usually a non‐thermal plasma, which can be better coupled with the catalyst. Therefore, the DBD reactor is more suitable for decomposing CH_4_ to produce H_2_ and C_2_ products.^[^ ^218^, ^219^, ^235^ ^]^ In addition, a series of reactors (GA, RGA, MW, RF, etc.) that can produce thermal plasma are used to decompose CH_4_ to generate carbon black materials. Thermal plasma has the characteristics of high energy density and high temperature, and the decomposition efficiency of CH_4_ is better. At the same time, these reactors can achieve high CH_4_ conversion without coupling with catalysts (Table 3).

### 3.2 PADRM

**Figure 5:**
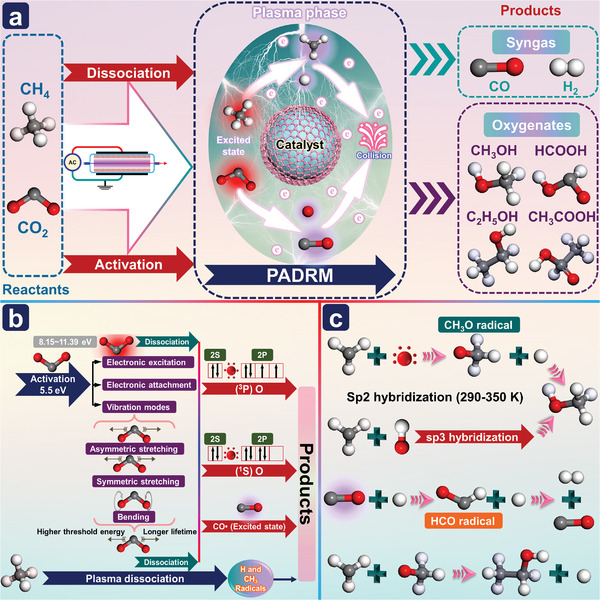
a) Schematic diagram of possible plasma‐assisted dry reforming of methane process; b,c) Possible elementary plasma‐chemical reactions in PADRM process.

The conversion of CO_2_ into value‐added chemicals has been regarded as a vital factor in creating a sustainable low‐carbon economy in the chemical and energy industries. Conventional DRM processes can convert CO_2_ and CH_4_ into syngas (CO and H_2_) and liquid oxygenates, due to thermodynamics limits, the latter is more difficult. Plasma‐assisted dry methane reforming (PADRM) is considered a potential method for converting CH_4_ and CO_2_ into syngas and high‐value chemicals under mild conditions (**Figure** **5**a), especially for distributed processes based on renewable energy.^[^ ^33^ ^]^

In the PADRM system, the elementary plasma‐chemical reactions of CO_2_ molecules are of interest. CO_2_ is a stable molecule with high chemical inertness, and its C—O bond strength and Gibbs free energy of formation are 532 and 394.6 kJ mol^−1^, respectively. Stability and chemical inertia lead to threshold energy of the direct dissociation of CO_2_ molecules as high as 11 eV. In a nonthermal plasma system, the number of electrons with such high kinetic energy is limited. Therefore, realizing the continuous induced direct dissociation of CO_2_ molecules is challenging.^[^ ^240^ ^]^ However, similar to the dissociation of CH_4_ molecules, the dissociation of CO_2_ molecules is performed stepwise. CO_2_ molecules are converted into excited CO_2_ by electron impact, excitation, and attachment in a nonthermal plasma, which can reduce the dissociation threshold energy of CO_2_ to 5.5 eV,^[^ ^31^ ^]^ when CO_2_ is added to the reactor, as shown in Figure 5b. Initially, collisions with electrons generate vibrationally excited CO_2_. There are three different vibrational modes for excited CO_2_: symmetric stretching, asymmetric stretching, and bending. Among these fundamental modes, asymmetric stretching has a longer lifetime and higher threshold energy and is the main channel for inducing the dissociation of excited CO_2_.^[^ ^241^ ^]^ During this vibrational excitation process, electron, and molecule collisions primarily excite the low vibrational levels of the ground electronic state. Next, the vibrational–vibrational relaxations lead to the dissociation of CO_2_ to produce CO and ground state oxygen atoms ((^3^P)O) with a lifetime of tens of microseconds.^[^ ^241^ ^]^ During this process, for some excited states of CO_2_, the excitation energy ranges between 8.15 and 11.39.^[^ ^242^ ^]^ This can induce the dissociation of CO_2_ because of the low threshold energy. Finally, CO_2_ dissociation occurs.^[^ ^243^, ^244^ ^]^ In addition, recent studies have demonstrated that thermal dissociation is the key mechanism for CO_2_ dissociation in a thermal plasma system.^[^ ^245^, ^246^, ^247^ ^]^ CO_2_ dissociates to produce CO and O radicals at temperatures higher than 3000 K (2726.85 °C) and further produces C and O radicals at temperatures higher than 6000 K (5726.85 °C).^[^ ^30^ ^]^ In thermal plasmas, CO_2_ molecules are often excited by vibrations, which leads to the dissociation of CO_2_.^[^ ^170^, ^240^ ^]^ However, this process requires a high vibrational–translational nonequilibrium.^[^ ^248^ ^]^ Additionally, O(^1^S) production channels can also be found during CO_2_ dissociation. If the energy is above 11.45 eV, the collisions could cause the dissociation of CO_2_ to CO(a^3^∏) metastable particles (kinetic energies are in the range of 0–1.2 eV). In addition, this mechanism produces a ground‐state O atom.^[^ ^249^ ^]^ Furthermore, during the thermal plasma process, the O radicals attach to electrons to form O^−^ radicals due to their electronegativity.^[^ ^19^ ^]^

Generally, in the plasma bulk, the electron collision reaction (i.e., electron collision excitation, dissociation, and ionization) is the main component of the plasma chemical reaction. The threshold energies for the direct dissociation of CH_4_ and CO_2_ are 9 eV and 11 eV, respectively.^[^ ^204^, ^249^ ^]^ In conventional plasmas, the density of electrons with such high kinetic energy is low, making it difficult to dissociate the CH_4_ and CO_2_ molecules directly. However, the dissociation of CH_4_ and CO_2_ occurs in steps, and the electron‐collision dissociation threshold energy of CO_2_ can be lowered (5.5 eV) if excited by electron‐collision electron excitation and electron‐collision vibrational excitation. According to previous studies, several electron collisions, including Reactions R3.17‐R3.23, lead to CO_2_ dissociation.^[^ ^241^, ^249^, ^250^, ^251^ ^]^

(R3.17) $$e+{\mathrm{CO}}_{2}\left(X^{1}\sum_{g}^{+}\right)\to e+{\mathrm{CO}}_{2}\left(1^{1}\sum_{u}^{+}\right)$$

(R3.18) $$e^{\prime }+{\mathrm{CO}}_{2}\left(1^{1}\sum_{u}^{+}\right)\to e+\mathrm{CO}\left(X^{1}\sum^{+}\right)+O\left({}^{1}S\right)$$

(R3.19) $$e+{\mathrm{CO}}_{2}\left(X^{1}\sum_{g}^{+}\right)\to e+\mathrm{CO}\left(a^{3}\prod \right)+O$$

(R3.20) $$\begin{array}{c} e+{\mathrm{CO}}_{2}\left(X^{1}\sum_{g}^{+}\right)\to e+\mathrm{CO}\left(a^{3}\prod \right)+O\left({}^{3}P\right)\;\left[E_{a}=11.45\;eV\right] \end{array}$$

(R3.21) $$\begin{array}{c} e+{\mathrm{CO}}_{2}\left(X^{1}\sum_{g}^{+}\right)\to e+\mathrm{CO}\left(a^{3}\prod \right)+O\left({}^{5}S\right)\;\left[E_{a}=20.6\;eV\right] \end{array}$$

(R3.22) $$e+{\mathrm{CO}}_{2}\left(X^{1}\sum_{g}^{+}\right)\to e+{\mathrm{CO}}_{2}\left(001\right)$$

(R3.23) $$e^{\prime }+CO_{2}\left(001\right)\to e+\mathrm{CO}\left(X^{1}\sum^{+}\right)+O\left({}^{1}S\right)$$

#### 3.2.1 Liquid Oxygenates Production via PADRM System

Synthesis of value‐added liquid oxygenates (CH_3_COOH, HCOOH, CH_3_OH, C_2_H_5_OH, etc.) by PADRM is less difficult than conventional DRM processes due to the characteristics of plasma chemistry far from thermodynamic equilibrium, and thus arousing great interest. To date, very limited efforts have been devoted to this challenging process–one‐step conversion of CH_4_ and CO_2_ to value‐added liquid oxygenates via the PADRM process. Krawczyk et al.^[^ ^252^ ^]^ performed PADRM reactions in the temperature range of 130–340 °C by using a DBD reactor. H_2_, CO_2_, hydrocarbons, and alcohols (CH_3_OH and C_2_H_5_OH) were detected in the reactor outlet stream, where ethane is the major product and the selectivity of liquid oxygenates is low. In contrast, Wang et al.^[^ ^253^ ^]^ developed a novel DBD reactor with a water electrode for the one‐step conversion of CO_2_ and CH_4_ to liquid oxygenates at room temperature (30 °C) and atmospheric pressure. The experimental results show that no reaction occurs in the “catalyst‐only” mode in the absence of plasma. However, the use of plasma enables this thermodynamically unfavorable reaction to occur at room temperature and produces liquid oxygenates including CH_3_COOH, CH_3_OH, C_2_H_5_OH, and HCHO, of which CH_3_COOH is the major product with a selectivity of 40.2%. The PADRM process shows great potential for manipulating the distribution of liquid oxygenates in a given process. Previous studies have indicated that there may be two reaction pathways for the formation of CH_3_COOH.^[^ ^254^ ^]^ I) Direct coupling of carboxyl radicals (COOH) and CH_3_ radicals,^[^ ^255^ ^]^ and COOH can be generated by the CO + OH addition or hydrogenation of CO_2_^−^.^[^ ^142^, ^253^, ^255^, ^256^, ^257^ ^]^ (II) The reaction of CH_3_ and CO radicals generates CH_3_CO, which further reacts with OH radicals to generate CH_3_COOH.^[^ ^258^ ^]^ Both CH_3_ and CO radicals are involved in the reactions of the above two pathways. Consequently, the balance between the CO and CH_3_ radicals is a vital factor in enhancing the selectivity and yield of CH_3_COOH. In addition, the DFT method proved that the generation of CH_3_COOH via pathway (I) is energetically more feasible than that via pathway (II).^[^ ^259^, ^260^ ^]^ In the plasma, the formation of COOH was promoted by the H atoms and OH radicals, which increased the corresponding end products.^[^ ^194^ ^]^ The reaction between the CH_3_ and COOH radicals occurred without any barrier, while the reaction of radical coupling between H and COOH needed to overcome an energy barrier of 23.4 kJ mol^−1^. Therefore, the production of CH_3_COOH is much easier than that of the other products in the PADRM process, which provides a novel strategy for producing CH_3_COOH.

As shown in **Table** **4**, Ni‐based catalysts are widely used in PADRM systems. Supporting Ni on metal oxide supports (Al_2_O_3_,^[^ ^261^, ^262^ ^]^ SiO_2_,^[^ ^263^ ^]^ MgO,^[^ ^264^ ^]^ etc.) is an effective strategy to enhance catalyst activity. Mahammadunnisa et al.^[^ ^265^ ^]^ synthesized a Ni/Al_2_O_3_ catalyst with Ni nanoparticles as the active component via a single‐step combustion method, and applied it to the PADRM process to produce syngas. Compared with the plasma‐only reactor, the H_2_ selectivity increased from 35.0% to 59.0% and the H_2_/CO ratio increased from 1.18 to 2.25 after the catalyst was introduced into the reactor, and the conversions of CH_4_ and CO_2_ were 49.0% and 30.0%. Surprisingly, in the PADRM system, the performance of the Ni/Al_2_O_3_ catalyst is better than that of the Pd/Al_2_O_3_ (noble metal‐based catalysts) catalyst, which is different from the traditional DRM process. Sentek et al.^[^ ^266^ ^]^ synthesized a Pd/Al_2_O_3_ catalyst and applied it to the PADRM process to produce syngas, the highest conversions of CH_4_ and CO_2_ were 50.9% and 28.1%, and the selectivity of CO and H_2_ were 40.0% and 29.0%. Using more advanced supports in combination with noble metals can further enhance catalyst activity. For example, Pt‐modified metal‐organic frameworks (MOFs) materials (PtNP@UIO‐67) have better syngas selectivity than UiO‐67 (the selectivity to hydrocarbons decreased by 30%, compared to the UiO‐67 packing).^[^ ^267^ ^]^ However, the high cost of noble metal‐based catalysts is an unavoidable problem, so bimetallic catalysts may be a better choice. From Table 4, the bimetallic catalysts Mo_2_C‐Ni/Al_2_O_3_ and Ni‐La/MCM‐41 have better catalytic activity and product selectivity than noble metal‐based catalysts and single‐metal catalysts in DBD plasma. It is worth mentioning that catalysts do not always improve CO_2_ and CH_4_ conversion in PADRM systems. Wang et al.^[^ ^253^ ^]^ found that Cu/Al_2_O_3_, Au/Al_2_O_3,_ and Pt/Al_2_O_3_ reduced the conversion of reactants due to the suppressed plasma discharge. These contradictory phenomena demonstrate the complexity of the PADRM system and the effect of catalysts on the reaction.

**Table 4:** Summary of the performance of various PADRM systems

Plasma action stage	Catalyst	Reactor types	Feed composition	Maximum conversion [%]	Selectivity of products [%]	Refs.
Catalytic	Ni/Al_2_O_3_	DBD	CH_4_:CO_2_ = 1:1	19.0 (CH_4_); 12.0 (CO_2_)	45.0 (H_2_); 22.0 (CO)	[261]
	Ni/AC	DBD	CH_4_:CO_2_ = 1:1	65.0 (CH_4_); 64.0 (CO_2_)	27.9 (H_2_); 32.5 (CO); 16.0 (C_2_H_6_)	[279]
	La_2_O_3_+ Al_2_O_3_	Corona	CH_4_:CO_2_ = 2:1	24.1 (CH_4_); 24.9 (CO_2_)	23.7 (CO); 68.2 (C_2_)	[280]
	PtNP@UiO‐67	DBD	CH_4_:CO_2_:Ar = 1:1:99	43.0 (CH_4_); 55.0 (CO_2_)	65.0 (H_2_); 63.0 (CO)	[267]
	Pd/Al_2_O_3_	DBD	CH_4_:CO_2_ = 2:1	50.9 (CH_4_); 28.1 (CO_2_)	29.0 (H_2_); 40.0 (CO); 9.45 (C_2_H_6_)	[266]
	Ag/Al_2_O_3_	DBD	CH_4_:CO_2_ = 2:1	52.0 (CH_4_); 29.0 (CO_2_)	37.9 (H_2_); 28.7 (CO); 7.5 (C_2_H_6_)	[266]
	Ni/SiO_2_	Glow discharge	CH_4:_CO_2_ :Ar = 1:1:2	70.0 (CH_4_); 68.0 (CO_2_)	98.0 (H_2_); 97.0 (CO)	[263]
	Ni/Al_2_O_3_	Glow discharge	CH_4_:CO_2_:Ar = 1:1:2	100.0 (CH_4_); 85.0 (CO_2_)	98.0 (H_2_)	[281]
	Cu/*γ*‐Al_2_O_3_	DBD	CH_4_:CO_2_ = 1:1	≈16.5 (CH_4_); ≈7.3 (CO_2_)	13.5 (CO); 40.2 (CH_3_COOH)	[253]
	Al_2_O_3_	DBD	CH_4_:CO_2_:Ar = 1:1:1	52.2 (CH_4_); 31 (CO_2_)	23.7 (H_2_); 18.7 (CO); 10.2 (C_2_H_6_)	[252]
	Fe/Al_2_O_3_	DBD	CH_4_:CO_2_:Ar = 1:1:1	45.9 (CH_4_); 20.4 (CO_2_)	20.8 (H_2_); 13.6 (CO); 9.1 (C_2_H_6_)	[252]
	NaY	DBD	CH_4_:CO_2_:Ar = 1:1:1	48.7 (CH_4_);19.3 (CO_2_)	21.2 (H_2_); 10.3 (CO); 5.7 (C_2_H_6_)	[252]
	Na‐ZSM‐5	DBD	CH_4_:CO:Ar = 1:1:1	65.1 (CH_4_); 40.1 (CO_2_)	21.3 (H_2_); 4.2 (CO); 6.7 (C_2_H_6_)	[252]
	CaO	DBD	CH_4_:CO_2_:He = 2:1:75	21.1 (CH_4_); 11.2 (CO_2_)	6.2 (H_2_); 30.2 (CO); 25.7 (C_2_H_6_); 4.8 (C_3_H_8_)	[282]
	Ni/Al_2_O_3_	NTP	CH_4_:CO_2_ = 2:1	52 (CH_4_); 43 (CO_2_)	56.3 (H_2_); 38.5 (CO)	[265]
	Ni–La/MCM‐41	RF	CH_4_:CO_2_ = 1:1	71.0 (CH_4_); 69.0 (CO_2_)	65.0 (H_2_); 73.0 (CO)	[283]
	AE‐NiO/*γ*‐Al_2_O_3_	DBD	CH_4_:CO_2_ = 1:1	70.8 (CH_4_);73.1 (CO_2_)	46.5 (H_2_); 44.8 (CO); 11.3 (C_2_H_4_)	[284]
	NF catalyst	DBD	CH_4_:CO_2_ = 1:1	17.9 (CH_4_); 80.3 (CO_2_)	53.9 (H_2_); 68.0 (CO)	[285]
	Mo_2_C‐Ni/Al_2_O_3_	DBD	CH_4_:CO_2_:Ar = 1:1:8	81.0 (CH_4_); 85.0 (CO_2_)	90.0 (H_2_); 85.0 (CO); 3.2 (C_2_); 1.5 (C_3_)	[286]
Noncatalytic	None	DBD	CH_4_:CO_2_ = 1:1	7.5 (CH_4_); 3.3 (CO_2_)	43.0 (H_2_); 60.0 (CO);15.0 (C_2_H_6_); 5.0 (C_3_H_8_)	[287]
		GA	CH_4_:CO_2_ = 3:7	13.1 (CH_4_); 8.4(CO_2_)	31.4 (H_2_); 69.5 (CO)	[154]
		DBD	CH_4_:CO_2_:Ar = 1:1:66	43.0 (CH_4_); 25.0 (CO_2_)	44.0 (H_2_); 54.0 (CO); 3.0 (C_2_H_6_)	[130]
		DBD	CH_4_:CO_2_ = 1:1	18.3 (CH_4_); 15.4 (CO_2_)	20.0 (CO); 33.7 (CH_3_COOH); 11.9 (C_2_H_5_OH);	
		DBD	CH_4_:CO_2_:Ar = 1:1:1	56.8 (CH_4_); 35.7 (CO_2_)	26.7 (H_2_); .2 (CO); 10.0 (C_2_H_6_)	[252]
		RF	CH_4_:CO_2_ = 1:1	55.0 (CH_4_); 30.0 (CO_2_)	25.0 (H_2_); 23.0 (CO)	[163]
		DBD	CH_4_:CO_2_ = 1:1	51.4 (CH_4_); 42.0 (CO_2_)	38.2 (H_2_); 43.2 (CO); 10.5 (C_2_H_6_)	[279]
		RGA	CH_4_:CO_2_ = 2:3	12.0 (CH_4_); 10.4 (CO_2_)	9.5 (H_2_); 9.5 (CO)	[59]
		DBD	CH_4_:CO_2_ = 4:5	45.0 (CH_4_); 40.0 (CO_2_)	95.0 (H_2_); 78.0 (CO)	[288]
		Glow discharge	CH_4_:CO_2_ = 20:7	64.0 (CH_4_); 94.0 (CO_2_)	35.0 (H_2_); 95.0 (CO)	[271]

DBD is the most popular type of plasma reactor used for PADRM processes (Table 4), mainly due to the simple structure of the DBD reactor, which makes it easy to scale up and industrial applications.^[^ ^33^ ^]^ Furthermore, the simple design allows packing catalysts and ferroelectric materials inside the reactor to improve the selectivity of the desired product. The reactant conversion and energy density of a DBD reactor are dependent on input power, reactor size, operating pressure, and temperature. The introduction of inert gases (such as He, Ar, and N_2_) and catalysts can improve the selectivity of syngas or different hydrocarbons.^[^ ^268^, ^269^ ^]^ In a DBD reactor, CO_2_ and CH_4_ are excited by high‐energy electron collisions when gas enters the discharge region. The most abundant species in DBD plasmas are vibrationally excited CH_4_, whose density is 100 times higher than that of free electrons.^[^ ^237^ ^]^ However, the plasma generated by DBD reactors is usually non‐thermal plasma, which suffers from the same problem of low energy density as corona discharge plasma and glows discharge plasma, which makes it rather difficult to achieve a higher conversion at a higher flow rate.^[^ ^189^ ^]^ However, reactors capable of generating thermal plasma are very expensive and consume more electricity. To overcome these problems, RGA discharge between thermal and nonthermal plasmas was introduced.^[^ ^59^ ^]^ The RGA reactor is a configuration that makes more efficient use of the energy, it has been reported that the RGA reactor can generate syngas at low temperature (475 °C) without any catalyst with a chemical efficiency (ratio of energy required to carry out the reaction at 25 °C to the used electrical energy) as high as 40%.^[^ ^193^ ^]^ However, the structure of the RGA reactor is relatively complex, the catalyst cannot be placed in the plasma area, and the temperature distribution inside the reactor is not uniform. In addition, RF,^[^ ^163^ ^]^ spark,^[^ ^270^ ^]^ PTP,^[^ ^165^ ^]^ and glow discharge^[^ ^271^ ^]^ plasma have been applied to the PADRM system, but the related literature is very limited, DBD is still the most promising reactor in PADRM system.

In most PADRM systems, the CH_4_/CO_2_ ratio in the feed is a key factor affecting reactant conversion and product distribution. In general, increasing the CH_4_/CO_2_ ratio decreases the conversion of CH_4_ while increasing the conversion of CO_2_.^[^ ^265^, ^272^ ^]^ This is believed to be since relatively less CO_2_ in the feed reduces the concentration of oxygen radicals that can attack CH_4_ molecules, thereby reducing CH_4_ conversion.^[^ ^267^ ^]^ Allah and Whitehead^[^ ^154^ ^]^ combined an RGA reactor with NiO/Al_2_O_3_ catalyst for dry methane reforming. The experimental results showed that increasing the CH_4_/CO_2_ ratio decreased the CH_4_ conversion, while the CO_2_ increased with the increase in the CH_4_/CO_2_ ratio. The CH_4_/CO_2_ ratio also affects SIE (specific input energy) and product selectivity.^[^ ^11^, ^271^ ^]^ Mahammadunnisa et al.^[^ ^265^ ^]^ found that syngas selectivity and SIE increased with decreasing CH_4_/CO_2_ ratio in PADRM system, H_2_ and CO selectivity were 35.0 and 38.0% (SIE = 5.2 kJ L^−1^), 32.5 and 38.5% (6 kJ L^−1^), 25.0 and 43.0% (7.2 kJ L^−1^) for 2:1, 1:1, and 1:2 CH_4_/CO_2_ ratios, respectively. When the CH_4_/CO_2_ ratio is too high, it will lead to an increase in the selectivity of C_2_ hydrocarbons.^[^ ^270^ ^]^ Moreover, some studies have shown that the carbon deposition in the PADRM reaction is also related to the CH_4_/CO_2_ ratio.^[^ ^19^, ^240^, ^273^ ^]^ In general, reducing the CH_4_/CO_2_ ratio can suppress the formation of carbon deposits, suggesting that excess CO_2_ is beneficial to prevent catalyst deactivation.^[^ ^31^ ^]^ Nikoo et al.^[^ ^274^ ^]^ used computational modeling to investigate the factors influencing carbon deposition in DRM systems. The calculation results show that carbon deposition decreases proportionally with the increase of CO_2_ content in the feed. When CH_4_/CO_2_ is around 1/3, no carbon deposits will be formed in the reaction system (>700 °C). In addition, the incorporation of noble gases into the feed also affects the efficiency of the PADRM system. Ramakers and Jo et al.^[^ ^275^, ^276^ ^]^ investigated the effect of noble gases on CO_2_ and CH_4_ conversion in DBD plasma. The results indicated that the addition of inert gas to the reactor gas can improve the conversion of CH_4_ and CO_2_. However, the energy efficiency of the system is reduced because a portion of the energy is used to ionize and excite the noble gas. Rahmani et al.^[^ ^130^ ^]^ found that the addition of Ar improved the discharge parameters of a DBD reactor. The number of micro‐discharges in the plasma area increased with the percentage of Ar in the mixed gas. When the Ar percentage of the Ar‐CH_4_‐CO_2_ mixture was increased from 0% to 66%, the plasma uniformity also increased for similar output power. Ar can achieve electronic excitation and ionization through inelastic collisions, and the corresponding activation energy thresholds were 11.55 and 15.76 eV, respectively, which were higher than those of CH_4_ and CO_2_. Therefore, the presence of Ar in the plasma phase reduced the frequency of inelastic collisions, resulting in higher electron energies. The electron density and temperature increased with increasing Ar content. As a result, a low breakdown voltage is sufficient to initiate plasma.^[^ ^277^, ^278^ ^]^ In summary, the efficiency of the entire plasma reaction system can be improved by adding noble gases during the PADRM reaction in the DBD reactor

### 3.3 PAPOM

In previous reports, PAPOM has been extensively studied using pure O_2_ as a coreactant.^[^ ^143^, ^289^ ^]^ However, excess O during the plasma chemical reaction can cause the complete oxidation of CH_4_ to CO_2_. Atomic oxygen produced in the air under certain plasma states is highly active in reacting with CH_4_ to generate oxygenates.^[^ ^290^ ^]^ Accordingly, using air instead of O_2_ as a co‐reactant is a more interesting and economical option for PAPOM. As shown in Reactions R3.24–R3.25, O_2_ in the air is the source of active oxygen species in the PAPOM reaction, which directly affects the dissociation process of hydrocarbons. These active oxygen species can further collide and react with other active particles in the plasma bulk, namely CH*_x_*, CO, H, and OH radicals, to produce the corresponding oxidation products, such as CO, CO_2_, CH_3_OH, and H_2_O (as in Reactions R3.26–R3.31).^[^ ^128^, ^135^, ^192^, ^290^, ^291^ ^]^

(R3.24) $$e+O_{2}\to e+2O$$

(R3.25) $$e+O_{2}\to 2e+O+O^{+}$$

(R3.26) $${\mathrm{CH}}_{4}+O\to {\mathrm{CH}}_{x}+\mathrm{OH}+\left(3-x\right)H$$

(R3.27) $${\mathrm{CH}}_{x}+\mathrm{OH}+\left(3-x\right)H\to {\mathrm{CH}}_{3}\mathrm{OH}$$

(R3.28) $${\mathrm{CH}}_{x}+O\to \mathrm{CO}+xH$$

(R3.29) $$H+H\to H_{2}$$

(R3.30) $$\mathrm{CO}+O\to {\mathrm{CO}}_{2}$$

(R3.31) $$H+\mathrm{OH}\to H_{2}O$$

**Figure 6:**
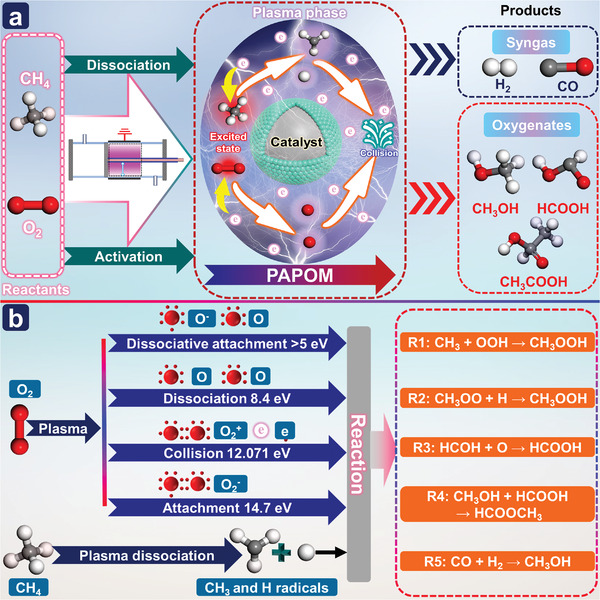
a) Schematic diagram of possible plasma‐assisted partial oxidation of methane process; b) Possible elementary plasma‐chemical reactions in PAPOM process.

The elementary plasma‐chemical reactions of O_2_ molecules in the PAPOM system are relatively simple. In a non‐thermal plasma system, an inelastic collision occurs between an electron with a certain energy and neutral oxygen molecules when oxygen passes through a high‐voltage discharge zone. In the plasma process shown in **Figure** **6**b, collisions by electrons generate vibrationally excited O_2_ (a^1^Δ_g_, b^1^Σ_g_^+^, Herzberg states, B^3^Σ_u_^−^), which can enhance the chain‐propagating reactions by CH_4_,^[^ ^292^, ^293^ ^]^ stimulating the production of active radicals and final products.^[^ ^294^ ^]^ Owing to its electronegativity, the O_2_ molecule easily forms negative ions in non‐thermal plasma by the way of dissociative attachment, attachment, and dissociation.^[^ ^194^, ^295^ ^]^ Finally, the O_2_ molecule is highly excited and dissociated to O [O (^3^P), O (^1^D)] via electron impact excitation and dissociation. In this process, energetic electrons transfer their kinetics to the molecules. In a thermal plasma system, the oxygen atoms generated by the direct dissociation of oxygen molecules are primarily in the ground state (^3^P) and metastable state (^1^D), and the required impact energies are 6.1 and 8.4 eV, respectively. The metastable state (^1^D) can be rapidly transformed to the ground state (^3^P) within 10 ns.^[^ ^77^, ^296^ ^]^ The inelastic collision of electrons with O_2_ molecules can directly affect the single (O^+^), double (O+ 2), and triple (O+ 3) ionization channels that can directly affect the plasma cracking process.^[^ ^128^, ^192^, ^293^ ^]^ In addition, electrons can impact the O_2_ molecule and metastable oxygen molecule O_2_(a^1^Δ_g_) to generate O+ 2, while O^+^ is generated by the electron impact ionization of O(^3^P), O(^1^D), and O_2_ pair creation.^[^ ^297^ ^]^

#### 3.3.1 Liquid Oxygenates Production via the PAPOM System

As shown in Figure 6b, the PAPOM process can generate multiple products. Conventionally, CH_4_ is converted to syngas by the SMR process, followed by gas‐liquid conversion to CH_3_OH, which is an indirect process, so direct partial oxidation of CH_4_ to CH_3_OH is highly desirable.^[^ ^298^, ^299^ ^]^ Giapis et al.^[^ ^300^ ^]^ applied for a patent on plasma microjet arrays for selective oxidation methane to CH_3_OH, claiming that this technology can produce CH_3_OH with high efficiency. Chawdhury et al.^[^ ^131^ ^]^ successfully achieved the partial oxidation of CH_4_ to CH_3_OH using a catalytic DBD plasma reactor. Typical results highlight the complementary effects of plasma and CuO/*γ*‐Al_2_O_3_ catalysts, where the introduction of CuO/*γ*‐Al_2_O_3_ catalysts in the DBD reactor significantly improves CH_4_ conversion and CH_3_OH selectivity. The selectivity to CH_3_OH was ≈34% after experimental conditions were optimized. The major dissociation intermediates were CH_3_ and O species in the plasma reactors. CH_3_OH, HCO, and HCOOH were formed in the micro/DBD plasma reactor. The direct dissociation of CH_4_ is difficult in a plasma environment.^[^ ^301^ ^]^ However, the addition of O_2_ can promote the dissociation of CH_4_, and O_2_ relatively easily forms negative ions in the discharge because of its electronegative nature. Active ionic oxygen species such as O^−^, O_2_^−^, and O_2_^2−^ are believed to initiate the dissociation of alkane C—H bonds to form CH*_x_*.^[^ ^302^ ^]^ These radicals can stably adsorb on the catalyst surface to form CH_3_OH according to the Langmuir‐Hinshelwood reaction. However, the generation of CH_3_OH via an indirect pathway (CH_3_→CH_3_O→CH_3_OH) is more energetically favorable because of the high stability of the adsorbed CH_3_ radicals.^[^ ^108^, ^110^, ^302^ ^]^ The above indirect pathway needs to satisfy the following conditions: I) the electron energy is not high enough or CH_3_ is the most abundant primary radical during the discharge process, and II) the reaction region contains enough ionic oxygen or O radicals to generate CH_3_O radicals, and III) low‐temperature oxidation is possible by quenching, not resulting in the kinetic path [CH_3_O → CH_2_O] but [CH_3_O → CH_3_OH].^[^ ^303^ ^]^ Accordingly, the DBD plasma reactor is suitable for POM to generate products of CH_3_OH (R3.32).

(R3.32) $$O_{2}+{\mathrm{CH}}_{4}\to 2{\mathrm{CH}}_{3}\mathrm{OH}\;\Delta H_{298K}=-78.44\;\mathrm{kJ}\;{\mathrm{mol}}^{-1}$$

Although the PAPOM system can overcome some inherent drawbacks of conventional catalytic processes, its selectivity is always lower than conventional processes, resulting in complex product distributions (**Table** **5**). A possible solution is to utilize plasma–catalyst coupling to improve the product selectivity of PAPOM.^[^ ^290^ ^]^ Chawdhury et al.^[^ ^131^ ^]^ used a CuO/*γ*‐Al_2_O_3_ catalyst combined with DBD plasma to generate CH_3_OH and determined that the formation of CH_3_OH did not follow the CH_3_+OH → CH_3_OH pathway; however, owing to insufficient energy, the extraction of H from CH_4_ by O_2_ is unlikely to form OH radicals. This is supported by the theoretical study of Starik et al.^[^ ^293^ ^]^ It is more likely to form intermediate CH_3_OO to generate CH_3_OH. Additionally, the presence of the CuO/*γ*‐Al_2_O_3_ catalyst improved the charge transfer, thereby increasing the CH_3_OH generation efficiency. In another study by Chawdhury et al.,^[^ ^135^ ^]^ it was revealed that at any voltage, a shorter discharge gap transferred more charge and led to the immediate formation of products such as HCHO and CH_3_OH. In addition, the selectivity and yield of H_2_ and C_2_H_6_ increased significantly with increasing residence time, and the selectivity of CO and CO_2_ exhibited an upward trend. Puliyalil et al.^[^ ^27^ ^]^ proposed HCOH formation from CH_3_OH oxidation at MoO_2_ or MoO_3_ catalyst surfaces in DBD, where Mo exhibits the critical behavior of changing its oxidation state and donating oxygen.

**Table 5:** Summary of the performance of various PAPOM systems

Plasma action stage	Catalyst	Reactor types	Reaction conditions	Maximum conversion [%]	Selectivity of products [%]	Refs.
Catalytic	Mo‐CuO/Al_2_O	DBD	CH_4_:Air = 1:1	36.0 (CH_4_)	3.5 (CH_3_OH); 15.0 (CO)	[304]
	Fe_2_O_3_	DBD	CH_4_:Air = 1:1	10.66 (CH_4_)	36.0 (CH_3_OH)	[290]
	Ni/Al_2_O_3_	Rotating electrode	CH_4_:O_2_ = 2:1	50.0 (CH_4_)	50.0 (H_2_); 2 (CO)	[295]
	Ni/MgO	DBD	CH_4_:O_2_:Ar = 4:2:94	100.0 (CH_4_)	80.0 (H_2_); 90 (CO)	[160]
	Cu‐Zn‐Al	DBD	CH_4_:O_2_ = 4:1	31.0 (CH_4_)	11.2 (H_2_); 35.7 (CO); 10.6 (CO_2_)	[289]
	CuO/*γ*‐Al_2_O_3_	DBD	CH_4_:O_2_ = 5:1	9.0 (CH_4_)	11.0 (H_2_); 37.0 (CH_3_OH); 6.5 (CO_2_); 5.3 (C_2_H_6_);	[131]
Noncatalytic	None	Pulsed spark discharge	CH_4_ + O_2_	41.0 (CH_4_)	78.0 (H_2_); 85.0(C_2_H_2_)	[305]
		DBD	CH_4_:O_2_ = 4:1	28.1 (CH_4_)	17.0 (H_2_); 45.5 (CO); 21.5 (CO_2_)	[289]
		Microplasma	CH_4_:O_2_ = 2:1	50.0 (CH_4_); 100 (O_2_)	15.0 (H_2_); 2.0 (CH_3_OH); 3.0 (HCOH); 1.0 (HCOOH); 25.0 (CO); 15.0 (CO_2_)	[302]
		RA	CH_4_:O_2_ = 1:2	>50.0 (CH_4_)	58.0 (H_2_); 5.00 (CO)	[118]
		DBD	CH_4_:O_2_ = 5:1	9.0 (CH_4_)	15.0 (H_2_); 37.0 (CH_3_OH); 6.0 (CO_2_); 9.8 (C_2_H_6_)	[131]

In thermal plasma, Jo et al.^[^ ^118^ ^]^ studied the effect of gas temperature in PAPOM. The results demonstrated that temperature has a limited effect on plasma chemistry over the evaluated temperature range. Moshrefi et al.^[^ ^295^ ^]^ evaluated the performance of a rotating ground electrode plasma reactor in the PAPOM reaction and showed that increasing the O_2_/CH_4_ molar ratio decreased the conversion. Using air instead of pure O_2_ increases the conversion of CH_4_ andO_2_ but reduces the selectivity to syngas (H_2_ and CO). Wang et al.^[^ ^192^ ^]^ used a GAD reactor to generate H_2_ via a PAPOM reaction. They determined that the collisions of high‐energy electrons and excited N_2_ species (mainly N_2_(A)) with other CH_4_ and O_2_ species in the plasma region are the two main pathways for activating this reforming system. This work was stimulated by Rafiq et al.^[^ ^157^ ^]^ used MATLAB software. The model simulation results were compared with the experimental data, and it was found that the calculated results of the conversion rates of CH_4_ and O_2_, the yields of H_2_ and CO, and the mole fraction of species were close to the actual experimental data. In thermal plasma (RA, RF), syngas were formed by the thermal dissociation of neutral CH_4_ and O_2_ molecules, and an almost 100% conversion rate was achieved.

### 3.4 PASMR

The Ni‐catalyzed steam methane reforming with natural gas as feedback is currently the dominant industrial technology for H_2_ production, and this process meets approximately 50% of the global demand for H_2_ because the components used have the largest mole fraction of H_2_.^[^ ^76^, ^90^ ^]^ Although SMR has been commercialized, the entire industrial process has struggled to cope with the recent demand for energy savings and emissions reduction, primarily because SMR is an energy‐intensive industry with low energy utilization efficiency.^[^ ^91^ ^]^ The plasma‐assisted steam methane reforming (PASMR), which is a targeted optimization of the conventional SMR process, is an emerging research topic. The PASMR process provides a unique method to induce a gas‐phase reaction, which has several important advantages, such as mild reaction conditions, compact reaction equipment, rapid response time, compatibility with a variety of hydrocarbons, and elimination of catalyst carbon deposition.^[^ ^124^ ^]^ In addition, the PASMR can effectively avoid greenhouse gas emissions. Another significant advantage of the PASMR is that it can be combined with other CH_4_ reforming routes to improve the CH_4_ conversion, product selectivity, and system efficiency, including combined plasma‐assisted SMR and DRM^[^ ^194^, ^306^ ^]^ and combined plasma‐assisted SMR and POM.^[^ ^97^, ^307^ ^]^ Combining PASMR with PADMR can provide two advantages compared with a single PASMR reaction: i) H_2_O provides additional O, thereby increasing the O/C ratio, which helps oxidize the soot; ii) H_2_O dissociation provides additional H atoms, thereby adjusting the H_2_/CO molar ratio in the product stream; and (iii) the catalyst has an improved coke‐resistance due to the introduction of H_2_O.^[^ ^138^ ^]^ At present, Shahverdi and Carabin^[^ ^308^ ^]^ have applied for relevant patents. Moreover, the combination of PASMR and PAPOM is beneficial to balance the heat load.^[^ ^97^ ^]^

**Figure 7:**
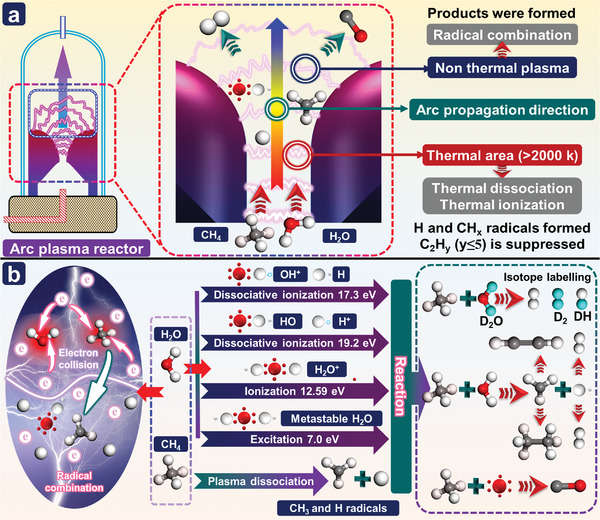
a) Schematic diagram of possible arc plasma‐assisted steam methane reforming; b) Possible elementary plasma‐chemical reactions in the PASMR process.

In a PASMR system, the elementary plasma‐chemical reactions of H_2_O molecules have a significant effect on the system efficiency. The relevant research based on the PASMR reaction mechanism and reaction products is summarized in Figure 6a. The H_2_O molecule has a V‐shaped structure with an angle of 104.5°, which gives the molecule a strong polarity. There are two O—H bonds in one H_2_O molecule, and the bond energy of each is 498.7 kJ mol^−1^. Generally, the threshold energy required to directly dissociate a single H_2_O molecule exceeds 7 eV.^[^ ^117^ ^]^ In a nonthermal plasma system, the process that dominates the initiation of H_2_O dissociation is electron collision excitation (**Figure** **7**b).^[^ ^309^, ^310^ ^]^ H_2_O molecules are first excited by collisions with high‐energy electrons. The excited H_2_O molecules are further dissociated in the plasma phase to produce H, OH, and other free radicals. Owing to the low mass of electrons, the energy loss of the electrons in the inelastic elastic collisions with H_2_O molecules is low. The collision of such high‐energy electrons with the H_2_O molecule breaks its chemical bonds, leading to the disintegration of the overall structure. After the H_2_O molecule is excited, more OH radicals and H atoms are formed. Xia et al.^[^ ^194^ ^]^ found that H atoms are easily formed during steam reforming because of the dissociation of OH radicals. H_2_O in thermal plasma requires high energy to sustain a stable plasma,^[^ ^311^ ^]^ which can maintain a stable atmosphere to generate the products. Generally, in a thermal plasma system, the dissociation of H_2_O occurs predominantly through the reaction: H_2_O→OH+H. The dissociation and recombination mechanisms of H_2_O in thermal plasma are extremely complex, primarily involving the dominant species H radicals, OH radicals, H_2_ radicals, O_2_ radicals, and H_2_O. In most cases, OH radicals can exist stably in thermal plasma. However, they can combine with H radicals when the plasma temperature decreases.^[^ ^311^ ^]^ Direct ionization requires threshold energy higher than 12.59 eV to form H_2_O^+^, higher than 17.3 eV to form OH^+,^ and 19.2 eV to form H^+^.^[^ ^117^ ^]^ Further increases in the electron energy open additional reaction channels. Therefore, in the PASMR system, H_2_O provides active species, namely H, OH, and O, through the dissociation reactions in the plasma bulk (R3.33–R3.35).^[^ ^312^ ^]^ These active species play a crucial role in the plasma chemical reactions,^[^ ^97^ ^]^ and they can activate CH_4_ by increasing the rate of CH_3_ radical formation to form other products (R3.36–R3.38).^[^ ^313^ ^]^ Accordingly, in the PASMR reaction, the CH_4_ conversion usually increases with the H_2_O content.

(R3.33) $$e+H_{2}O\to H_{2}+O$$

(R3.34) $$e+H_{2}O\to H+\mathrm{OH}$$

(R3.35) e+OH→H+O+e

(R3.36) $${\mathrm{CH}}_{4}+\mathrm{OH}\to {\mathrm{CH}}_{3}+H_{2}O$$

(R3.37) $${\mathrm{CH}}_{4}+H\to {\mathrm{CH}}_{3}+H_{2}$$

(R3.38) $${\mathrm{CH}}_{4}+O\to {\mathrm{CH}}_{3}+\mathrm{OH}$$

As shown in **Table** **6**, the introduction of catalyst can indeed improve the conversion of reactants and the selectivity of synthesis gas in the PASMR system. However, unlike the numerous catalysts for the traditional SMR process, the catalysts developed for the PASMR process are very limited. In fact, from the literature statistics, the catalyst‐free PASMR system seems to be more popular. Zhu et al.^[^ ^88^ ^]^ developed a PASMR device for distributed H_2_ production, for which thermal plasma generated by the GAD reactor initiates the reaction, followed by Ni/CeO_2_/Al_2_O_3_ in a heat‐insulated reactor without extra heating. For the coupled plasma‐catalytic process, the conversion is close to the thermodynamic equilibrium value and thus has complete selectivity for CO and CO_2_. This coupled process achieves a CH_4_ conversion rate of 90% under optimal conditions with higher energy efficiency (75%) and energy cost (1.5 kWh Nm^−3^) than the plasma‐only process (59% and 2.3 kWh Nm^−3^). A similar phenomenon was also observed by Liu et al.^[^ ^314^ ^]^ These results demonstrate that plasma‐catalytic coupling is a feasible strategy to improve the efficiency of PASMR systems, and more research is needed in the future.

**Table 6:** Summary of the performance of various PASMR systems

Plasma action stage	Catalyst	Reactor types	Reaction conditions	Maximum conversion [%]	Selectivity of products [%]	Refs.
Catalytic	Ni/CeO_2_+Al_2_O_3_	GAD	CH_4_:H_2_O = 1:3	90.0 (CH_4_); 42.0 (H_2_O)	100.0 (H_2_); 52.0 (CO); 46.0 (CO_2_)	[88]
	Ni/Al_2_O_3_	DBD	CH_4_:H_2_O = 1:3	68.0 (CH_4_)	69.0 (H_2_); 15.1 (CO); 6.1 (CO_2_)	[318]
	Ni/Al_2_O_3_	DBD	CH_4_:H_2_O = 2:1	45.8 (CH_4_)	100.0 (H_2_); 40.0 (CO); 60.0 (CO_2_)	[314]
Non‐catalytic	None	GAD	CH_4_:H_2_O = 1:3	65.0 (CH_4_); 21.0 (H_2_O)	96.0 (H_2_); 77.0 (CO); 10.0 (CO_2_)	[88]
		Arc	CH_4_:H_2_O = 1:1	99.5 (CH_4_)	99.9 (H_2_)	[124]
		DBD	CH_4_ + H_2_O + N_2_;	46.0 (CH_4_)	92.0 (H_2_); 22.0 (CO); 5.0 (C_2_H_4_)	[314]
		MW	CH_4_:H_2_O = 1:1	92.0 (CH_4_)	92.7 (H_2_); 16.3 (C_2_H_2_);	[73]
		DBD	CH_4_:H_2_O:N_2_ = 1:1:8	6.4 (CH_4_); 1.8 (H_2_O)	45.0 (H_2_); 28.6 (C_2_H_6_); 18.0 (CO)	[313]
		RF	CH_4_ + H_2_O	40.0 (CH_4_)	95.0 (H_2_); 20.0 (CO)	[159]
		MW	CH_4_+H_2_O	51.4 (CH_4_)	42.1(H_2_); 3.3 (CO); 0.4 (C_2_H_4_); 5.0 (C_2_H_2_),	[151]
		MW	H_2_O:CH_4_ = 6:1	95.0 (CH_4_)	63.4 (H_2_); 10.4 (CO); 6.3 (CO_2_)	[317]

Different from the other three plasma‐assisted methane reforming routes (PAMD, PADRM, and PAPOM) in which the DBD reactor is the mainstream, there are more reports on the application of RGA, MW, and RF reactors that can generate thermal plasma in the PASMR system. This may be because methane steam reforming is an endothermic process, and although the plasma can change its energy equation, it cannot bypass the potential endothermicity, thus requiring a higher temperature from the plasma reactor. Liu et al.^[^ ^313^ ^]^ investigated the relationship between the plasma temperature and product selectivity and yield when using a DBD reactor for PASMR reactions. Electron‐induced chemistry (i.e., reduced electric field intensity and discharge power) and thermochemistry (i.e., background gas temperature) control the transformation of reactants during plasma chemistry. With increasing plasma temperatures, thermochemistry has a positive effect on the CH_4_ conversion and a negative effect on the H_2_O conversion, whereas electron‐induced chemistry has little effect on product distribution. However, thermochemistry can have a strong impact on product selectivity. Zheng et al.^[^ ^312^ ^]^ used CHEMKIN‐PRO software to simulate a non‐thermal plasma process. These results indicate that the recombination of CH_3_ radicals is the limiting reaction for the generation of CO, and O is a vital species for CO generation. Montoro‐Damas et al.^[^ ^315^ ^]^ used isotope labeling to investigate the H_2_O reforming of CH_4_ in a DBD reactor using D_2_O and CH_4_ as reactants. The results indicated that different partitions of H and D atoms were found in the reactants, and H_2_ production was detected using mass spectrometry. This demonstrated that the plasma formation of intermediate excited species is inefficient for the formation of H_2_ products. In thermal plasma, Choi et al.^[^ ^150^ ^]^ conducted PASMR reactions using MW reactors and found that MW plasmas could provide highly reactive species and high‐temperature plasma flames, which could achieve high chemical reaction rates without catalysts. Wang et al.^[^ ^73^ ^]^ found that steam inhibited the formation of C_2_H_2_, thus improving the selectivity of H_2_ in MW plasma reactors. The decrease in C_2_H*_x_* selectivity was approximately equal to the increase in CO_2_ selectivity, whereas the CO selectivity was constant. Putra et al.^[^ ^159^ ^]^ used RF plasma to generate H_2_. They proposed that the H_2_ selectivity was determined by the number of H atoms in the H_2_ content of the product gases. The GAD reactor is also suitable for the PASMR route to generate H_2_.^[^ ^88^ ^]^ However, in arc plasma reactors, regions far from the arc discharge cannot effectively participate in the reaction, which also causes different temperatures in different regions. The inhomogeneous temperature distribution in the reactor inhibits the formation of C_2_H*_x_* hydrocarbons (which can lead to coking); however, it also increases the product selectivity, which is also consistent with previous literature.^[^ ^119^, ^316^ ^]^

Likewise, the H_2_O/CH_4_ ratio has a significant effect on the conversion of H_2_O and CH_4_ as well as on the product distribution, although the SMR stoichiometric reaction shows that 1 mol of H_2_O (g) is required to reform 1 mol of CH_4_, in practice, the reaction starts with higher H_2_O/CH_4_ ratios were performed. Typically, conventional SMR processes are performed at an H_2_O/CH_4_ ratio of about 3 to avoid coking on the catalyst and to obtain maximum H_2_ yield.^[^ ^80^ ^]^ Choi et al.^[^ ^150^ ^]^ reported a catalyst‐free microwave plasma SMR technology for H_2_ production, the H_2_ selectivity at H_2_O/CH_4_ = 3 is 71.3% and the CH_4_ conversion is 95.3%. Akande and Lee^[^ ^317^ ^]^ found that the H_2_O/CH_4_ ratio is the vital factor affecting the CH_4_ conversion, with CH_4_ conversion close to 100% under given experimental conditions and increasing with H_2_O/CH_4_ ratio. However, when the H_2_O/CH_4_ ratio exceeds 4, CH_4_ conversion showed no significant conversion; this implies a reduction in the energetic parameters in the discharge zone. Zhu et al.^[^ ^88^ ^]^ found that with the H_2_O/CH_4_ ratio increase, the formation of C_2_H*_x_* was inhibited and WGS was promoted. As the H_2_O/CH_4_ ratio increased from 1.5 to 3, the CH_4_ conversion increased from 46% to 55%, while the H_2_O conversion decreased from 27% to 17%. This is attributed to the forward shift of the SMR reaction equilibrium as the reactant H_2_O concentration increases. Due to the low concentration of C_2_H*_x_* in the products, the H_2_ selectivity did not change significantly, the selectivity of C_2_H*_x_* decreased by 2% (from 4%), and the selectivity of H_2_ increased slightly by 2% (to 98%).

### 3.5 Development of Catalysts for PARM System

Research on plasma catalytic CH_4_ reforming is a new topic, and various catalysts, including Ni‐based, iron‐based, noble metal‐, and carbon‐based catalysts, have been applied to the PARM reaction. Through comparison and screening, most current research has focused predominantly on the development of Ni‐based catalysts. The participation of catalysts can effectively improve the conversion of CH_4_ and reduce the reaction energy barrier of CH_4_ reforming by deriving the intermediate products. Because the catalyst can adsorb dissociated reactants or some intermediate products, it is always favorable for the conversion of CH_4_. In the field of CH_4_ reforming, there are two routes of combining plasma and catalysis: plasma‐modified catalysts and plasma synergistic catalysis. This section briefly introduces catalyst applications in the PARM process and discusses possible directions for catalyst development.

#### 3.5.1 Plasma‐Modified Catalyst for CH_4_ Reforming

Plasma pretreatment of the catalysts can improve their activity by improving the physical and chemical properties of the surface. The main mechanism involves changing the microscopic morphology of the catalysts, increasing their porosity, and exposing more active sites through the bombardment and etching of high‐energy electrons. Furthermore, the acid‐base and redox properties of the catalyst can be improved by introducing functional groups on the surface of the catalyst.^[^ ^27^, ^319^ ^]^ Plasma‐modified catalysts will lead to slower growth of the active center metal, reducing the particle size and increasing the exposed area of the active center, enabling the modified catalyst to exhibit low‐temperature activity.^[^ ^320^ ^]^ These metal nanoparticles formed by slow growth will moderately reduce the specific rate of CH_4_ decomposition, thus making the process smoother than the solid carbon to gaseous carbon process, which can inhibit the deposition of carbon to a certain extent.^[^ ^321^ ^]^ Notably, the plasma‐modified supporter destroys the surface structure to provide more defects to anchor the active metal atoms, and the increase in defects leads to better dispersion of the active center under the condition of fixed active metal loading.^[^ ^322^ ^]^ This is beneficial for CH_4_ conversion and reducing coking. Liu et al.^[^ ^320^ ^]^ used glow discharge plasma to treat the Pd/HZSM‐5 catalyst, which showed higher catalytic activity and higher stability compared to the catalyst without plasma treatment. At 450 °C, the CH_4_ conversion on the plasma‐treated catalyst was close to 100%, which was much higher than that of the non‐plasma‐treated catalyst (≈50%). Odedairo et al.^[^ ^323^ ^]^ found that the plasma pretreatment process prevented the tendency of Ni atoms to migrate into the bulk of supports and avoid the diffused interfacial region, hence, the plasma‐treated samples exhibited good stability and high catalysis in the DRM reaction. In addition, catalysts such as Ni–Cu/Al_2_O_3_, NiMgSBA‐15, and Ni/Al_2_O_3_ also showed better performance in the methane reforming process after plasma treatment.^[^ ^323^, ^324^, ^325^ ^]^ These phenomena strongly demonstrate plasma‐modified catalysts' great potential in the methane reforming field. Plasma technology has been widely used for catalyst/adsorbent modification and has been proven to be an efficient, fast, and easy‐to‐operate material pretreatment technique.^[^ ^18^, ^120^ ^]^ There are relatively few reports of plasma‐modified catalyst technology in the field of methane reforming, but it has promising prospects.

#### 3.5.2 Plasma–Catalyst Interactions in PARM Systems (Plasma Catalysis)

**Figure 8:**
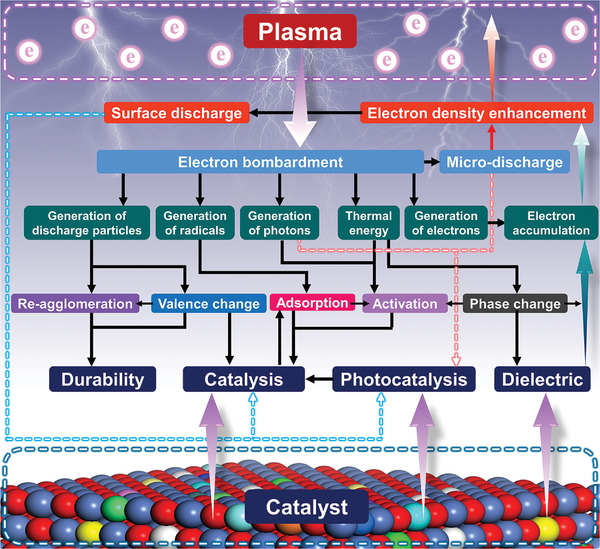
Possible interactions between catalyst and plasma in PARM system. Reproduced with permission.^[^ ^31^ ^]^ Copyright 2016, Elsevier.

It is well known that the C—H bond in CH_4_ molecules is difficult to activate,^[^ ^326^ ^]^ whereas H_2_O and CO_2_ molecules are also very stable. The coactivation of CH_4_ and other reactant molecules via different CH_4_ reforming routes is challenging. Due to thermodynamic limitations, the CH_4_ reforming reaction usually proceeds at high temperatures (≈800 °C).^[^ ^327^, ^328^ ^]^ Although many advances and significant achievements have been made in high‐temperature CH_4_ reforming, the development of catalysts for PARM reactions has been relatively scarce. In addition, when no catalyst was placed in the plasma reactor, the conversion of reactants and selectivity of the products were generally low. The interaction between the plasma and catalyst reduces the kinetic energy barrier related to conventional high‐temperature catalysis and high‐pressure treatment and greatly reduces the energy cost.^[^ ^329^ ^]^ Owing to the complexity of the coupled plasma–catalysis system, special attention must be paid to distinguishing the plasma‐induced adsorption and desorption processes on the catalyst surface and the unwanted side reactions and desired reactions, which are crucial for improving the efficiency of the reaction system. The possible synergistic effects between the catalyst and plasma in the PARM system are presented in **Figure** **8**. The dissociated atoms in the plasma are highly electrophilic, and the electron‐rich centers adsorbed on the catalyst surface easily react and desorb.^[^ ^330^ ^]^ Therefore, the surface reactions within the plasma reactor are more complex than conventional thermal catalysis. Specifically, the plasma establishes a strong electric field, which generates highly reactive species by dissociating the gas molecules. The plasma can also improve the properties of the catalyst, such as surface area, morphology, and oxidation state,^[^ ^170^ ^]^ thereby improving the activity of the catalyst. When the catalyst exists in the plasma phase, charge accumulation and polarization can be achieved by enhancing the electric field and promoting the formation of microdischarges on the rough surface and pores of the catalyst, thus increasing the adsorption rate and contact time of the active species, thereby enhancing the possibility of collisions and reactions.^[^ ^331^, ^332^ ^]^ In addition, when the plasma and catalyst are coupled, the CO selectivity can be improved by suppressing the formation of CH*_n_* on the catalyst surface.^[^ ^333^, ^334^ ^]^ In the study, the catalyst could be placed in the plasma region (DBD) or downstream of the plasma (MW and GA) depending on the geometry of the plasma reactor, discharge pattern, plasma temperature, and thermal stability of the catalyst.^[^ ^55^ ^]^ Placing the catalyst in the plasma region is not feasible for thermal plasmas (e.g., MW torches, RF torches, and gliding arcs) because of the high plasma temperature (thousands of °C). The two‐stage combination is a more reasonable option for thermal plasma‐assisted methane reforming.

Nonetheless, until further development of novel catalysts for PARM systems, it is essential to clarify whether plasma assists catalysis or catalysis assists plasma. There is significant debate in the literature about the plasma–catalytic mechanisms of methane reforming. Chen et al.^[^ ^61^ ^]^ believed that this question should be answered from the perspective of CH_4_ conversion. Taking SMR as an example, the typical operating temperature of this reaction process is between 700 °C and 900 °C. The energy required to increase a CH_4_ molecule from 25 °C to the above temperature range is between 0.38 and 0.53 eV. In nonthermal plasmas, CH_4_ molecules are mainly transformed by dissociation and ionization, with minimum threshold energy of 9.0 eV. Therefore, in the nonthermal plasma‐assisted catalytic reforming of the CH_4_ system, the conversion of reactants is mainly contributed by thermal catalysis, whereas the nonthermal plasma excites the reactant activity and provides a highly reactive reaction environment. However, Mehta et al.^[^ ^60^ ^]^ summarized the application of non‐thermal plasma‐driven catalysis in DRM processes and pointed out that non‐thermal plasma‐catalyzed DRM processes are dominated by plasma driven. Furthermore, some research groups report that CH_4_ conversion is nearly independent of bulk temperature in plasma‐only and plasma–catalytic processes when the temperature is 350–550 K (77–277 °C).^[^ ^266^, ^335^, ^336^ ^]^ Instead, the CH_4_ conversion increased with increasing SEI, indicating that the plasma activation of CH_4_ drove the reaction.

The selection of suitable catalysts is crucial for PARM reactions. Ni‐based catalysts are mainstream research of PARM catalysts, while non‐Ni‐based catalysts include doped noble metals, transition metals, and bimetallic catalysts.

#### 3.5.3 Ni‐Based Catalysts

Benefiting from the advantages of low cost and high activity, Ni‐based catalysts can be applied to almost all plasma‐assisted methane reforming situations, including different reforming routes (PAMD, PADRM, PAPOM, and PASMR) and reactors (DBD, MW, RGA, RF, etc.). To enhance the activity of Ni‐based catalysts, a common strategy is to support Ni active components on advanced supports with high specific surface area and high stability, supports of interest include Al_2_O_3_,^[^ ^272^, ^337^ ^]^ La_2_O_3_,^[^ ^338^ ^]^ CeO_2_,^[^ ^339^ ^]^ ZrO_2_,^[^ ^340^ ^]^ MgO,^[^ ^341^ ^]^ SiO_2_,^[^ ^263^ ^]^ and molecular sieves.^[^ ^342^ ^]^ In most cases, the introduction of Ni‐based catalysts in PARM systems can significantly improve the conversion of reactants. For example, Wang et al.^[^ ^343^ ^]^ studied the Ni/*γ*‐Al_2_O_3_ in DBD plasma for PADRM. The conversions of CH_4_ and CO_2_ are only 11% and 21% in the plasma‐only case. After introducing the Ni/*γ*‐Al_2_O_3_ catalyst into the reaction system, the conversions of CH_4_ and CO_2_ were increased to 33% and 22.5%. Ni‐based catalysts can also significantly improve the product selectivity of the PARM system, Tao et al.^[^ ^344^ ^]^ performed the PADRM experiments under plasma‐only and plasma‐catalyzed synergistic conditions, respectively. Under the action of a commercial Z107 Ni/Al_2_O_3_ catalyst, the PADRM system can achieve higher CH_4_ and CO_2_ conversion and higher H_2_ and CO selectivity. The conversions of CH_4_ and CO_2_ were 96.33% and 84.63%, and the selectivity of CO and H_2_ was 91.99% and 74.23%. These values are 10–20% higher than the plasma‐only situation. Moreover, many authors have emphasized that the activity of Ni‐based catalysts in PARM systems follows the following rules: the better the Ni dispersion and the smaller the Ni particle size, the better the catalyst activity.^[^ ^126^ ^]^ Some studies have shown that the combination of two different supports gives Ni‐based catalysts higher CH_4_ conversion and better product distribution. Nagaraja et al.^[^ ^345^ ^]^ found that double‐support catalysts (Ni‐MgO‐ZrO_2_) had better conversion of CH_4_ (80%) and CO_2_ (88%) than single‐support catalysts (Ni‐MgO and Ni‐ZrO_2_) during PADRM process. The high CO_2_ conversion is ascribed to the strong basic nature of double‐support and high active metal (Ni) dispersion, owing to the addition of MgO. The addition of promoters to nickel‐based catalysts can also improve metal dispersion and enhance metal‐support interactions. The addition of strongly basic oxides such as CaO and MgO also increases the basicity of the catalyst, thereby promoting CO chemisorption and CH_4_ activation.^[^ ^346^ ^]^

#### 3.5.4 Non‐Ni‐Based Catalysts

Noble metals, transition metals, and bimetallic are also common catalysts in PARM systems. Noble metals (Pt, Pd, Ag, etc.) are usually used as active components or catalyst promoters, from previous studies, noble metal‐promoted catalysts exhibit strong anti‐coking properties and high catalytic activity in different PARM systems.^[^ ^267^ ^]^ For instance, Hu et al.^[^ ^235^ ^]^ successfully prepared stable and highly dispersed Pd on CeO_2_ supports for DBD plasma‐assisted methane decomposition. The strong interaction of Pd with the CeO_2_ support effectively avoided the sintering of the active components during the high‐temperature catalytic test. The synergistic effect of Pd/CeO_2_ can effectively avoid the generation of carbon deposition. Pd‐based catalysts can also significantly alter the product distribution of PARM, which may be related to the hydrogenation reaction on the Pd surface.^[^ ^141^, ^266^, ^280^ ^]^ Ghorbani et al.^[^ ^347^ ^]^ tested the conversion and product selectivity of different metal catalysts (Ni, Fe, Pd, and Cu) for the decomposition of CH_4_ under plasma synergy. The experimental results show that Pd and Ni are effective catalysts for PAMD. Ni and Pd catalysts showed the highest yields and selectivity for propane and ethane, respectively. Pt‐ and Ag‐based catalysts have similar properties to Pd‐based catalysts in the PARM system. Pt, as a classical metal active component, has received attention in the field of PARM, while Ag is relatively less studied, and in some studies, the performance of Ag is weaker than that of Pd.^[^ ^266^ ^]^ However, due to the high cost of noble metals, the number of literature is much lower than that of Ni‐based catalysts. Recently, bi‐metallic catalysts prepared using combinations of noble and non‐noble metals have also been attractive, often combining high activity with anti‐coking properties. Khalifeh et al.^[^ ^219^ ^]^ used Pt‐Re/Al_2_O_3_ catalyst for DBD plasma PADRM and reported 100% conversions for CH_4_ (H_2_ selectivity of 100% and maximum energy efficiency of 26.08%), which also showed excellent coking resistance during reforming. Under certain conditions, inexpensive non‐nickel transition metal catalysts can also exhibit surprising performance in PARM systems. For instance, Wang et al.^[^ ^253^ ^]^ recently demonstrated that the PADRM system can produce oxygenated liquid products with high selectivity by carefully selecting catalyst and operating conditions. A water jacket was used as the external electrode to remove the resistive heat generated by the plasma and to keep the reaction temperature around 30 °C. The experimental results showed that the oxidation selectivity of the plasma‐only system to liquid products is 50–60%, in which acetic acid is the main component. The selectivity for these oxygenates greatly exceeds the trace levels reported in DBD reactors without external cooling, where the bulk temperature is 100 °C or higher.^[^ ^266^, ^269^ ^]^ The selectivity to acetic acid increased as the catalyst (Cu/*γ*‐Al_2_O_3_) was introduced into the plasma reactor. This work demonstrates that the rational design of catalysts can improve the selectivity of PARM systems to desired products.

A detailed introduction is difficult because of the large amount of literature and the variety of catalysts used. To date, no general rule has been established yet for the catalyst selection for PARM systems.^[^ ^27^ ^]^ Fortunately, several reviews have summarized and introduced PARM catalysts. Li et al.^[^ ^55^ ^]^ believe that scientific insights, including active catalytic sites and physicochemical properties, can be revealed through more advanced in situ characterization techniques and computer simulation techniques, and accordingly, efficient catalysts with high selectivity can be rationally designed. Chen et al.^[^ ^61^ ^]^ emphasized that the appropriate plasma source in a PARM system depends on the structure of the plasma catalytic system. Nonthermal plasmas are suitable for use with single‐stage plasma‐catalytic systems, whereas thermal plasmas are more suitable for use with two‐stage plasma‐catalytic systems. Chung and Chang^[^ ^31^ ^]^ demonstrated that catalysts could affect the performance of PADRM through several aspects: the photocatalysis effect, increasing electron density, and reducing the reaction temperature. In addition, the catalyst design should consider matching the type of reactor, Nguyen et al.^[^ ^148^ ^]^ proposed that the MW absorption capacity of the catalyst should be considered when using an MW plasma reactor for CH_4_ reforming. Carbon‐based catalysts such as graphene‐supported metal single‐atom catalysts (SACs) have been widely used in this field because of their strong MW absorption capacity. However, the disadvantage of carbon is that it is inevitably consumed by reacting with reactants (i.e., O_2_, CO_2_, and H_2_O). Metal‐based catalysts may generate strong MW reflections. The composite concept offers an attractive avenue for developing metal‐based catalysts because they allow the combination of highly active components with magnetic components capable of absorbing MW. Suitable non‐metallic supports can also be considered to solve the problem of sintering active components during MW irradiation. Molten metal/salt catalysts, which are brilliant in the conventional methane reforming process,^[^ ^45^, ^48^, ^348^ ^]^ are also an option worth considering for PARM systems. Molten alloy/salt catalyst has excellent carbon deposition resistance and activity and is suitable for combination with a thermal plasma system. However, there is no relevant report at present.

## 4 Summary and Outlook

Methane reforming is considered one of the major challenges of this century. Over the past two decades, some new technologies have made substantial progress. This paper presents an overview of the PARM process and its related plasma reactors that generate valuable products. The greatest feature of plasma technology is providing a high concentration of active species, thereby creating a highly reactive environment.^[^ ^32^ ^]^ Thus, reactions far from thermodynamic equilibrium also have the potential to occur, which is the intrinsic reason for the strong attraction of PARM. This review aims to inform readers of a previously incompletely understood emerging technology with a state‐of‐the‐art and critical assessment of PARM. Plasma‐based CH_4_ reforming is a relatively new and rapidly developing field, and like any emerging technology, it has much room for improvement and new (interdisciplinary) developments. Consequently, every part of PARM technology needs to be thoroughly studied and evaluated to make PARM commercially viable. Although there has been an increasing amount of literature and patents on PARM in recent years, we must acknowledge that PARM is still in its early stages. To close the gap with conventional methane reforming technologies, PARM systems still need to overcome these practical challenges:

1) The first challenge is the low selectivity of the PARM system for the desired products. At present, many reactors (such as Arc, DBD, DC, MW, and RGA) can achieve high CH_4_ conversion.^[^ ^124^, ^217^, ^219^, ^317^ ^]^ However, since the complexity of plasma chemistry, most PARM systems have low selectivity to desired products and complex product species distribution, limiting PARM systems' practicality.^[^ ^151^, ^302^ ^]^ Product selectivity is affected by many factors, and constructing catalysts with high selectivity is an effective strategy, however, so far, catalysts applied to PARM systems are mostly designed based on conventional methane reforming catalysts (Ni‐based catalysts), which are not optimally suited for plasma synergy. The light generated by the plasma is a non‐negligible factor, so the catalyst applied in the PARM system should be activated by visible light (or ultraviolet light). The literature on the application of catalysts with photocatalytic activity to PARM systems is very limited, and this research direction needs to be expanded. In addition, the structure‐activity relationship of the catalyst, size selection, packing method, placement position in the reactor, etc., are all issues that require further study.
2) The second challenge is the poor understanding of the nature of plasma related to nonequilibrium thermodynamics in PARM reactions.^[^ ^18^ ^]^ Since many plasma reactions (such as scattering, excitation, attachment, ionization, and dissociation of reactant molecules, sputtering, etching, deposition of catalyst, and several others) can occur simultaneously in a PARM system, careful control of reaction parameters such as energy density, electron energy distribution function, electron temperature, pressure, species flux and reaction time to control reaction kinetics better and avoid plasma damage to catalysts and electrodes. Furthermore, most existing equipment cannot directly measure active particles' species, density, and energy, which further increases the difficulty of exploring the reaction mechanism and kinetics.
3) The third challenge is to change the academic view of plasma technology and its applications, which is generally regarded as a high‐energy‐consuming technology and is only suitable for specific fields. The large‐scale commercial application of PARM system must consider the overall cost, and improving the system's energy efficiency is one of the solutions (currently in the 12–15% range).^[^ ^33^ ^]^ In the future, electricity costs are expected to decrease significantly owing to the development of renewable energy sources such as solar energy. Advances in plasma technology have led to further improvements in energy efficiency, in which case PARM system costs are expected to be significantly reduced.

**Figure 9:**
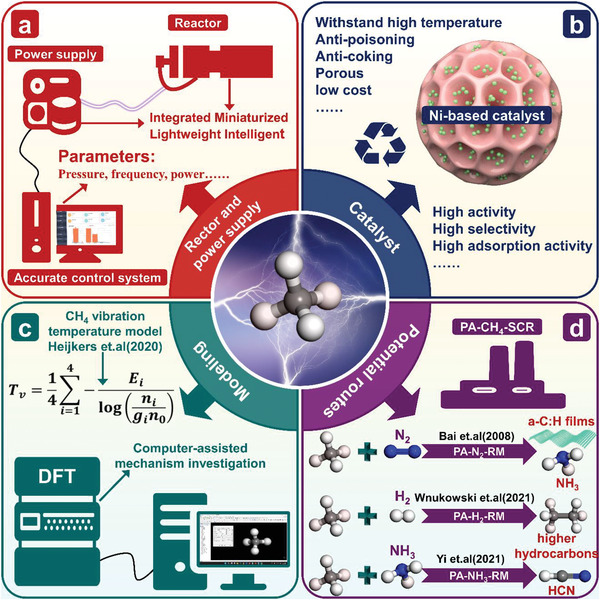
An outlook for the development direction of plasma‐assisted methane reforming. a) Design of efficient and powerful power supplies and reactors and match them with accurate control systems; b) construction of high activity, high selectivity, and anti‐coking Ni‐based catalysts; c) computer‐assisted PARM reaction mechanism investigation; d) more possible PARM routes.

Additionally, in the PARM system design process, it is necessary to carefully match the power supply and the reactor, that is, the type, structure, material, and size of the reactor, the control system, topology, and related components of the power supply, and to determine the optimal output parameters, such as frequency, voltage, power, and energy efficiency ratio. We must obtain a rational and in‐depth understanding of PARM to guide the design of the reaction process better. Based on the above understanding, we look for follow‐up research on PARM based on four aspects: reactor and power supply, catalyst, modeling, and potential PARM routes, as shown in **Figure** **9**.

### 4.1 Reactor and Power Supply

The reactor type and configuration offer various opportunities to improve the energy efficiency of the PARM system, whereas modifying the electrode geometry and its catalyst coating may enhance the discharge behavior. The reactor type and configuration affect the discharge characteristics and further influence the discharge parameters. Additionally, the electrode geometry inside the plasma reactor is also critical for determining the actual performance of the PARM system, which can directly affect the matching degree of the reactor and power supply, methane reforming effect, and energy utilization efficiency.^[^ ^349^, ^350^ ^]^ Miao et al.^[^ ^351^ ^]^ investigated the effects of various electrode geometries on discharge uniformity, power deposition, energy efficiency, and operating temperature. Taking the arc plasma as an example, the 3D gliding arc plasma reactor has a higher contact probability between the reactive gas and arc than the 2D arc technology. As a result, compared with the 2D arc configuration, 3D arc technology is more popular in industrial applications. The geometry of the plasma reactor and internal electrodes can significantly impact the operating efficiency of the PARM system. Therefore, before designing a plasma reactor, the relationship between the geometric parameters of the reactor and the PARM process should be fully considered. Furthermore, in different reactor configurations, the CH_4_ reforming reaction mechanism may be altered by the induced electric field, thereby affecting the reaction process, and changing the product distribution in the PARM reaction. Although some plasma reactors exhibit good performance, the available data and perspectives remain limited. Therefore, further research is required to fully exploit their potential. In the PARM reaction, the choice of reactor type and structural design needs to be determined according to the actual demand and CH_4_ reforming route. There is no perfect reactor, and only the right one.

A well‐matched power supply system to the reactor is a vital part of the PARM system, which determines the possibility of application of the PARM on an industrial scale and requires special design and construction methods.^[^ ^352^, ^353^ ^]^ The correct operation of the PARM system depends on the characteristics of the power supply system.^[^ ^354^ ^]^ In addition, the power supply system needs to respond promptly and accurately to the unusual receiver of the plasma reactor. An excellent plasma generation system should be able to effectively avoid electromagnetic interference, including conducted and radiated interference and surges. Consequently, the regulation of the output parameters (e.g., power, voltage, and frequency) over a wide range, good operational stability, high energy utilization efficiency, and anti‐interference are the most desirable features of the plasma reactor power supply system. The power supply of the plasma reactor is usually classified as a direct current (DC) or alternating current (AC). The mode of AC discharge usually includes power frequency and high‐frequency discharges. AC power supply adds a switching circuit to achieve the output pulse amplitude, pulse width, and pulse frequency based on the DC power supply. The number of pulse outputs can be adjusted and set according to the power supply requirements. Although an AC power supply has these advantages, its design and construction still need to be further optimized and improved. A typical example is the application of AC power to a DBD reactor, which is traditionally excited by AC power sources and operates in filamentary mode, which may result in inhomogeneous discharge, local overheating, and low energy efficiency.^[^ ^355^ ^]^ Hence, pulsed power sources (DC) have been used to excite DBD, which discharges more homogeneously. As a result, DC power can significantly improve energy efficiency and avoid the reactor overheating. Overall, a highly integrated, miniaturized, lightweight, and even intelligent plasma power supply system is a clear development trend, which requires the common progress of the entire industrial chain.

### 4.2 Catalyst

Improving the conversion of reactants and selectivity of products is a significant challenge in developing PARM, which may be overcome by plasma‐catalysis coupling.^[^ ^33^ ^]^ However, placing the catalysts in the plasma area may distort the electric field, change the discharge parameters, and weaken the performance of the reactor. These phenomena have been observed in many plasma systems (particularly in DBDs).^[^ ^19^ ^]^ Therefore, it is necessary to design and construct new well‐matched catalysts for the plasma–catalytic coupled reaction systems. One of the greatest advantages of plasma processing is its highly active environment. However, research on long‐term exposure of the catalyst to the plasma environment is lacking. It has been reported that the plasma environment can lead to overheating of catalysts or reorganization of active components.^[^ ^356^, ^357^ ^]^ Long‐term studies on the interaction between the plasma and catalyst are required before the large‐scale implementation of PARM systems. It is important to emphasize that in any coupled plasma–catalysis process, the complex surface and gas‐phase chemistry correlate strongly with the physical properties of the plasma and catalyst. Studying the reaction mechanisms and transformation processes in such complex systems is challenging and may require the development of new methods and techniques. For these reasons, it is difficult to study the plasma‐catalytic coupling processes in PARM with the application of complex catalytic systems. Consequently, simplifying the catalyst design and composition may help to understand PARM reactions. In addition, the coordination between reactor design and catalyst performance (surface reaction) is also a non‐negligible factor for the conversion of CH_4_ into desirable products.

Ni‐based catalysts have obvious advantages in PARM systems, but the carbon deposition of the active species inevitably leads to catalyst deactivation. For the PASMR, coke formation can lead to catalyst deactivation, especially when the carbon forms carbon filaments. Filamentous carbon materials have high mechanical strength, which causes the mechanical deformation of the catalyst.^[^ ^358^, ^359^ ^]^ Rare earth elements, such as Pr and La, have been used in PASMR or PADRM using Ni catalysts because Pr and La promote the electron conversion between Ni and the supporter, thus keeping Ni in a lower valence state. The doping of rare earth elements in Ni‐based catalysts can activate CO_2_, resulting in the formation of CO and O, which is very helpful in removing surface carbon species.^[^ ^360^ ^]^ It is essential to use a carrier to control the active species and prolong the life of the catalyst. CeO_2_ has excellent redox properties and self‐decoking properties owing to its very easy reaction with carbon species due to its lattice oxygen.^[^ ^361^, ^362^, ^363^ ^]^ If the catalyst has a strong interaction between Ni and the support, the anticoking performance can significantly improve. It is worth mentioning that the L acid sites in many supports, such as Na/ZSM‐5, will seriously deposit carbon. The oxides significantly increase the conversion to CH_4_.^[^ ^364^ ^]^ Therefore, the choice of O species‐labile metal oxides as supports (CuO or Fe*_x_* O*_y_*, CeO_2_) is favorable for PAPOM. However, these carriers may seriously interfere with the product selectivity in the PADRM and PAMD pathways because of their reactive O species. Furthermore, it is generally accepted that highly dispersed active centers are a vital requirement to prevent carbon deposition and sintering of active components (based on reported data in various PARM reactions). Therefore, loading Ni nanoparticles on stable supports with a high specific surface area is an effective strategy to develop efficient anti‐sintering catalysts. Core–shell catalysts (Ni nanoparticles as the core) and single‐atom catalysts not only improve metal sintering and avoid carbon deposition but also offer great prospects for high CH_4_ conversion and high product selectivity. Nonetheless, the major disadvantage of these catalysts is scaling up the synthesis process from laboratory to industrial scale. Therefore, developing economical and stable synthetic strategies is mandatory for linking all of the reported advantages of Ni‐based catalysts to the PARM system. In addition, non‐Ni‐based catalysts also deserve attention. Although noble metals inevitably bring high costs when used as active components of catalysts, when used as promoters of transition metal‐based catalysts, it is possible to strike a balance between cost, activity, and anti‐coking. Bi‐metallic catalysts and inexpensive transition metal catalysts also show satisfactory catalytic activity and product selectivity in some specific systems,^[^ ^253^, ^280^ ^]^ but whether they are universal to most PARM systems still needs further research. The molten metal/salt catalyst used in the conventional methane reforming process combines high activity and anti‐coking,^[^ ^46^, ^47^ ^]^ and the performance of this catalyst in the PARM system (especially thermal plasma) is expected. We must recognize that plasma‐catalysis is a multiparameter process and the high coupling between system parameters must be rationally understood. It is therefore necessary to systematically measure the influence of all electrical, mechanical, chemical, physical, gas dynamics, and thermodynamic parameters on the results. All the above work will provide help for the design of new plasma‐catalytic reactors and the construction of new catalysts, so as to have an in‐depth understanding of how these processes can be scaled up and commercialized.

### 4.3 Modeling

It is clear from this study that the product selectivity and CH_4_ conversion of different plasma reactors using the same CH_4_ reforming route are vastly different. However, the reasons for this phenomenon have not been studied in detail, and the underlying mechanisms are not completely understood. Because many unanticipated reactions may occur simultaneously in the plasma bulk, better optimization of reaction kinetics is required by precise control of reaction parameters (e.g., energy density, electron temperature, and species flux) to control the reaction in the target direction.^[^ ^365^ ^]^ However, achieving the above goals requires a deeper understanding of plasma physiochemistry, the interaction of plasma and gas molecules, and the reaction kinetics in the plasma environment, which are currently lacking. The highly complex and dynamic reaction environment brings extra difficulties to the in situ characterization of reaction systems, functional identification of plasma species, and monitoring of reaction paths, which also makes computational modeling difficult and time‐consuming, and only a few studies have proposed models based on the assumption of local thermodynamic equilibrium.^[^ ^366^ ^]^ Modeling the PARM reaction system is crucial for the analysis of CH_4_ reforming efficiency. This complex behavior of plasmas makes it challenging to connect the plasma species, chemical reaction pathways, and products. Nonetheless, to better understand the PARM reaction system, a better understanding of the macro‐ and microdynamics and fluid models of PARM is required. These are invaluable for optimizing existing PARM systems, studying the effects of discharge parameters on these processes, and guiding catalyst construction. A critical step in modeling PARM should include conducting detailed uncertainty analyses and sensitivity studies, which can reveal the impact of these uncertainties on the PARM model predictions and determine the accuracy of the model. Although this will be a significant work, it is necessary to continue along these routes because it is essential to fully explore the predictive characteristics of this model. Owing to the strongly unstable nature of the plasma coupled with its confinement within the plasma region, it is exceptionally difficult to directly observe the full dynamics of the plasma. Therefore, it is reasonable to use sufficient models in future studies to enhance our understanding of the kinetics of the plasma chemical reactions. Modeling PARM is extremely challenging because its flow is 3D, unstable, and nonlinear in nature and needs to be described on a wide range of temporal and spatial scales. Furthermore, the effects of chemical and thermodynamic nonequilibrium must also be considered, particularly near the boundaries of the plasma area; therefore, the minimum requirement for the PARM dynamics description is a 3D transient model. All these considerations may help to improve the energy utilization efficiency and product selectivity of PARM, thereby enhancing its economic viability and ultimately promoting its industrial application.

### 4.4 Potential PARM Routes

As the plasma system is far from thermodynamic equilibrium and provides extremely high concentrations of active chemicals, it can overcome the limitations of conventional methane reforming routes and offer more possibilities in chemical raw material production, environmental protection, energy storage, and material preparation. According to reports, plasma‐catalytic NH_3_ reforming of CH_4_ can synthesize hydrocyanic acid (HCN).^[^ ^367^, ^368^ ^]^ HCN is a valuable chemical raw material, and its industrial methods mainly include Andrussow and BMA processes, which are carried out at temperatures above 1000 °C. While the synthesis of HCN by C—N coupling reaction between CH_4_ and NH_3_ in plasma can reduce the reaction temperature to 400°C.^[^ ^369^ ^]^ In addition, plasma‐assisted N_2_ reforming of CH_4_ can synthesize NH_3_ and amorphous hydrogenated carbon (a‐C: H) films under mild conditions.^[^ ^197^, ^210^ ^]^ In contrast, plasma‐assisted H_2_ reforming of CH_4_ enables the selective synthesis of higher hydrocarbons.^[^ ^152^ ^]^ Another interesting application is the selective catalytic reduction of NO*_x_* by CH_4_ (CH_4_‐SCR) over a wide temperature range (250−550°C) using plasma technology.^[^ ^370^, ^371^ ^]^ Based on the above examples, we believe that there are many untapped routes for PARM. When expanding the application of PARM in the future, it is necessary to strictly evaluate the system operation efficiency, technical and economic issues, and environmental impact. With a clearer understanding of various process principles, PARM can work in a sustainable, economical, and efficient way to combat the greenhouse effect and produce the desired products.

In the future, a perfect solution to many of the issues brought forward is likely to rely on the true convergence of technology and solutions. Each technology and solution has its specific tasks, rather than the “one solution (or technology) fits all” mentality.^[^ ^170^ ^]^ From the current analysis, it is clear that PARM is still far from perfect industrialization. PARM is undoubtedly a CH_4_ reforming technology with great potential despite the various challenges. This paper only introduces a small part of many existing research results, but we hope readers will find that PARM is an interesting and constantly improving technology, and many exciting advancements and achievements can be expected in the coming years. We also encourage readers to join this fast‐growing field to advance the basic research further to realize the industrialized application of PARM, which requires multidisciplinary and interprofessional cooperation. Based on the firm belief in the great potential of PARM technology, we believe that PARM may be (at least) part of the dawn on the horizon.

## Conflict of Interest

The authors declare no conflict of interest.
